# Body condition scoring facilitates healthcare monitoring in Hermann’s Tortoises (*Testudo hermanni ssp*.)

**DOI:** 10.1371/journal.pone.0301892

**Published:** 2024-04-18

**Authors:** Julia Frankenberger, Jean-Marie Ballouard, Sebastien Caron, Yury Zablotski, Petra Kölle

**Affiliations:** 1 LMU Small Animal Clinic, Centre for Clinical Veterinary Medicine, LMU Munich, Munich, Germany; 2 Station d’Observation et de Protection des Tortues et de leurs Milieux (SOPTOM), CRCC, Gonfaron, France; Marine Science Centre, University of Basrah, IRAQ

## Abstract

Clinical assessment of body condition is crucial in captive and free-ranging reptiles, since a large percentage of diseases result from inadequate nutrition. However, preventive health care is restricted by the lack of a practical method for the assessment in tortoises. Pre-existing evaluation systems based on weight and shell measurements are laborious and ignore the clinical presentation of the animal. The present study aimed to facilitate the assessment by establishing a body condition score. A total of 373 Hermann’s Tortoises (*Testudo hermanni*) (n = 281 tortoises kept as pets in Germany and n = 92 tortoises originating from a free-ranging population (68) or a rearing station (24) in France) were examined and data (weight (g), carapace length (cm), width (cm), height (cm)) were recorded in a standard protocol between October 2020 and October 2021. A modified version of a body condition score for Mojave Desert Tortoises (*Gopherus agassizii*) (1 = cachectic, 3 = ideal, 5 = obese) was utilized and tested against pre-existing shell measurement systems (Jackson’s ratio, body condition index, volume condition index, circumferential product). German captive tortoises were significantly heavier and larger than French specimens. In the Spearman’s correlation matrix, the body condition score showed a statistically significant correlation with all measurement methods in the total population of captive tortoises (*Testudo hermanni boettgeri*), with a medium correlation strength, and a lack of correlation in free-ranging tortoises (*Testudo hermanni hermanni*). However, individual animal data suggested misleading results of mathematical equations in terms of body condition. Clinical evaluation of tortoises, including a body condition score, should be considered essential to provide good healthcare and should be an integral part of general examination.

## Introduction

While the Hermann’s Tortoise (*Testudo hermanni*) is likely Europe’s most popular reptile species in captivity [1, 2], it is listed as “Near threatened” in the IUCN Red list of endangered species [3]. Tortoise protection centres such as “Station d’Observation et de Protection des Tortues et de leurs Milieux” (SOPTOM) in the south of France are essential to secure their conservation in the wild [4]. Despite their popularity and conservation efforts, the fundamental assessment of their body condition continues to be challenging for veterinarians and ecologists. Given the large impact of diseases resulting from inadequate nutrition, clinical assessment of body condition in reptiles is crucial [5–7]. However, due to the particular difficulties posed by tortoise anatomy, preventive health care is restricted by the lack of a practical method of assessment.

Body condition, commonly described as a marker for the body’s energy reserves, is generally considered an important gauge of an animal’s health and fitness [8, 9]. It indicates the gross trophic and hydric status of individuals and is thus a key integrative parameter that responds to annual and environmental fluctuations in tortoises [10–12]. Recognizing high body condition as a sign of chronic energy surplus is an important step in preventing obesity-related diseases such as steatosis, yolk coelomitis and dystocia [7, 13]. Similarly, detecting poor body condition is crucial, as this can be a sign of malnutrition or an underlying chronic disease that threatens survival, especially during the annual hibernation period [14–17].

For the past few decades, the mathematical relationship between body mass and carapace length has been widely used as a non-destructive method of assessing growth and body condition in tortoises. This relationship was mainly used in the context of Jackson’s ratio [18, 19] and the body condition index [20] (Table 1). These methods, however, have raised concerns as the body mass of a tortoise may be influenced by many underlying factors, including the shape of the carapace, bone structure, bladder filling, state of reproduction in female specimens, gut content, potential lithophagy, oedema, or liver disease [21–23]. More recent considerations base their calculations on parameters such as shell volume instead of carapace length [24–27] (Table 1). These volume equations aim to eliminate factors as sexual dimorphism or interpopulation differences and meet increasing interest in the design of current body condition studies [26–28]. Suggestions for further simplification of these calculations were made by tortoise breeders [29, 30], with the approach of creating a formula from a circumferential product using rounded carapace measurements. While all of the above-mentioned equations present the assessor with an objective result and cause only minimal disturbance to the animal, the mathematic approach is too laborious for daily practice and ignores the clinical presentation of the animal itself.

**Table 1:** Summary of measurement methods for body condition assessment in tortoises used in the present study.

Method	Parameters	Formula
**Body condition score**	Muscle mass, fat tissue and bones	Subjective visual and palpatory assessment of muscularity and fat storage according to fixed skeletal points
**Jackson‘s ratio^a^**	Weight and carapace length	$\frac{weight(g)}{straight\;length{\left(cm\right)}^{3}}$
**Body condition index^a^**	Weight and carapace length, adjusted to sex and month	$\frac{LOG(weight(g))}{\left(-a+b*LOG(straight\;carapace\;length(mm)\right)}$ *-a and b are adjusted according to sex and month*
**Volume index based on a rectangle^b^**	Weight and carapace length, -height and -width	$\frac{weight(g)}{straight\;length*straight\;width*straight\;height(cm)}$
**Volume index based on an ellipsoid^b^**	Weight and carapace length, -height and -width	$\frac{6*weight(g)}{\pi *straight\;length*straight\;width*straight\;height(cm)}$
**Circumferential product^b^**	Weight and circumference of carapace length and -width	$\frac{weight(g)}{rounded\;length*rounded\;width(cm)}$

^a^In case of the Jackson’s ratio and the body condition index, the names were derived from literature

^b^The other systems were given descriptive names to facilitate discussion.

In this respect, the body condition scoring system (BCS) could provide a rapid and non-invasive measurement system, analogous to domestic mammals and zoo animals, where it already constitutes an integral part of health screening [31–39]. In general, the body condition score is a subjective, semi-quantitative instrument based on key skeletal elements, using both visual aspects of the body’s shape as well as palpatory features, such as the prominence of musculature, fat tissue and bones. The two most frequently applied BCS systems are classified on a scale of 1–5 or 1–9, with a low score (1–2 resp. 1–4) indicating under-condition, a middle score (3 resp. 5) assuming ideal condition and a high score (4–5 resp. 4–9) indicating over-condition. In tortoises, however, the shell hides notable features such as the ribcage or the body silhouette. In addition, in reptiles, adipose tissue is also stored in the liver and solid fat bodies in the coelomic area and not evenly subcutaneously, as their need for insulation is lower than in homeothermic animals [13, 40–42].

Recently, promising efforts have been made to create modified BCS systems for reptiles. Snakes such as Burmese pythons (*Python bivittatus*) or Corn snakes (*Pantheropis guttatus*) would seem to portray ideal candidates for validation of body condition assessments due to their simple body plans [43, 44], and American Crocodiles (*Crocodylus acutus*) and Leopard Geckos (*Eublepharis macularius*) also allow for full body examination [45, 46], however, the shell of chelonians poses a major constraint for the direct transfer of results, even within Testudines. The plastron of green turtles (C*helonia mydas*) may permit for a certain expansion of the body circumference and therefore allow for a degree of visual depiction of the fat storage within the body [47]. African Side-neck turtles display a wider inguinal field for mobility in water, therefore present the assessor with a larger visual area for examination [15]. There is little data on body condition scoring in tortoises. While Lamberski [48] provides an excellent collection of sample photographs and a detailed body condition protocol in their study on Mojave Desert Tortoises, however, no comparison with an objective measurement assessment was performed for its validation. Gimmel et al. [49] evaluated 34 Hermann’s Tortoises using two assessment points, which did not correlate with the Jacksons’ ratio or the body condition index.

The knowledge on body condition scoring in tortoises is largely based on very limited data. The aim of the present study was thus to propose a species specific BCS system to the Hermann’s Tortoise and test it against the above-mentioned objective calculations of shell measurement systems in a larger population (Table 1). Additionally, the study intended to evaluate how the factors carapace deformation, sex, origin, and subspecies may influence the correlation, and to what extend the shell measurement systems could therefore be substituted for one another. Ultimately, the study aimed to improve health screening in Hermann’s Tortoises by assessing whether the body condition score could provide a rapid, non-invasive, and low-cost general examination tool for domestic tortoises in veterinary practice and facilitate body condition assessment in free-ranging tortoises.

## Materials and methods

### Animals and husbandry

Most data originated from German captive tortoises. Tortoise breeders, private owners and reptile rescue stations in Germany participated in the study program following an appeal in Facebook groups or via direct phone or e-mail inquiries. This way, a total of 256 Hermann’s Tortoises (117 male and 139 female) kept as pets were assessed from October 2020 to October 2021. To reduce measurement variations, the same veterinarian conducted the assessments listed further below using the same equipment. Data was recorded in entry forms and later manually transferred into Excel sheets. Included were all clinically healthy *T*. *hermanni*, except for those missing extremities. A number of tortoises were also photographed to allow for later analysis. In addition, the French tortoise conservation centre “Station d’Observation et de Protection des Tortues et de leurs Milieux” (SOPTOM) were provided with a protocol including precise measurement guidelines and sample photographs. They submitted data on 27 male, 39 female and 2 unsexed Hermann´s tortoises originating from a free-ranging population in southern France and 6 male and 18 female Hermann’s tortoises from their breeding centre in Gonfaron in October 2021. Finally, data on 25 unsexed tortoises were provided by a German veterinary practice specialized in reptiles following the same protocol. All animals had a straight carapace length greater than 10 cm, as suggested by Stubbs et al. [50] and used in the study design by Willemsen and Hailey [20]. This resulted in a total study population of 373 Hermann’s Tortoises (Table 2).

**Table 2:** Hermann’s Tortoises measured in the present study, divided according to the respective criteria.

	German Tortoises (n)	French Tortoises (n)	Total study population (n)
**All sexes**	281	92	**373**
**Female**	139	57	**196**
**Male**	117	33	**150**
**Unsexed**	25	2	**27**
**T. h. hermanni**	25	92	**117**
**T. h. boettgeri**	256	0	**256**
**Captive**	281	24	**305**
**Free-ranging**	0	68	**68**

First, history was taken from the animal’s owner, and documents of the animal were sighted if available. Age was noted in years. If age could not be determined based on the documents, which was often the case with found animals in rescue stations, the age was estimated from growth rings on the plastron and carapace and the ventral midline seam of the plastron [51–53]. Accordingly, the animals were divided into corresponding age groups: 0 = under 5 years of age; 1 = 5–10 years of age; 2 = 10–15 years of age; 3 = 15–20 years of age; 4 = 20–30 years of age; 5 = 30–60 years of age; 6 = over 60 years of age. The husbandry of most tortoises could be inspected, and the feeding regime was noted. Animals measured from private owners, breeders, and rescue centres were kept outdoors, hibernated annually, and fed over 80% wild herbs and forage at the time of data collection.

Two subspecies of *Testudo hermanni* are recognized: *Testudo hermanni hermanni* Gmelin, 1789, which originates from the western Mediterranean region, and *Testudo hermanni boettgeri* Mojsisovits, 1889, whose range mainly comprises the Balkan region [54, 55]. For the distinction between these subspecies, identification papers and external features were used, such as the colour and shape of carapace and plastron, presence of a yellow cheek patch and the length of plastron seams. Most of the tortoises measured in Germany were animals of the subspecies *T*. *h*. *boettgeri*, while all French tortoises belonged to the subspecies *T*. *h*. *hermanni*.

### Body weight and metrics

As tortoises tend to defecate and urinate when handled, animals were treated carefully, and body mass was the first parameter recorded. An electronic scale (Tristar Electronic Balance®, Germany) was used for all measurements, and body mass was recorded to the nearest 1g. Sex was determined by plastron concavity, the shape of the rear marginal scute, position of cloaca and relative tail size [53, 56, 57].

**Fig 1:**
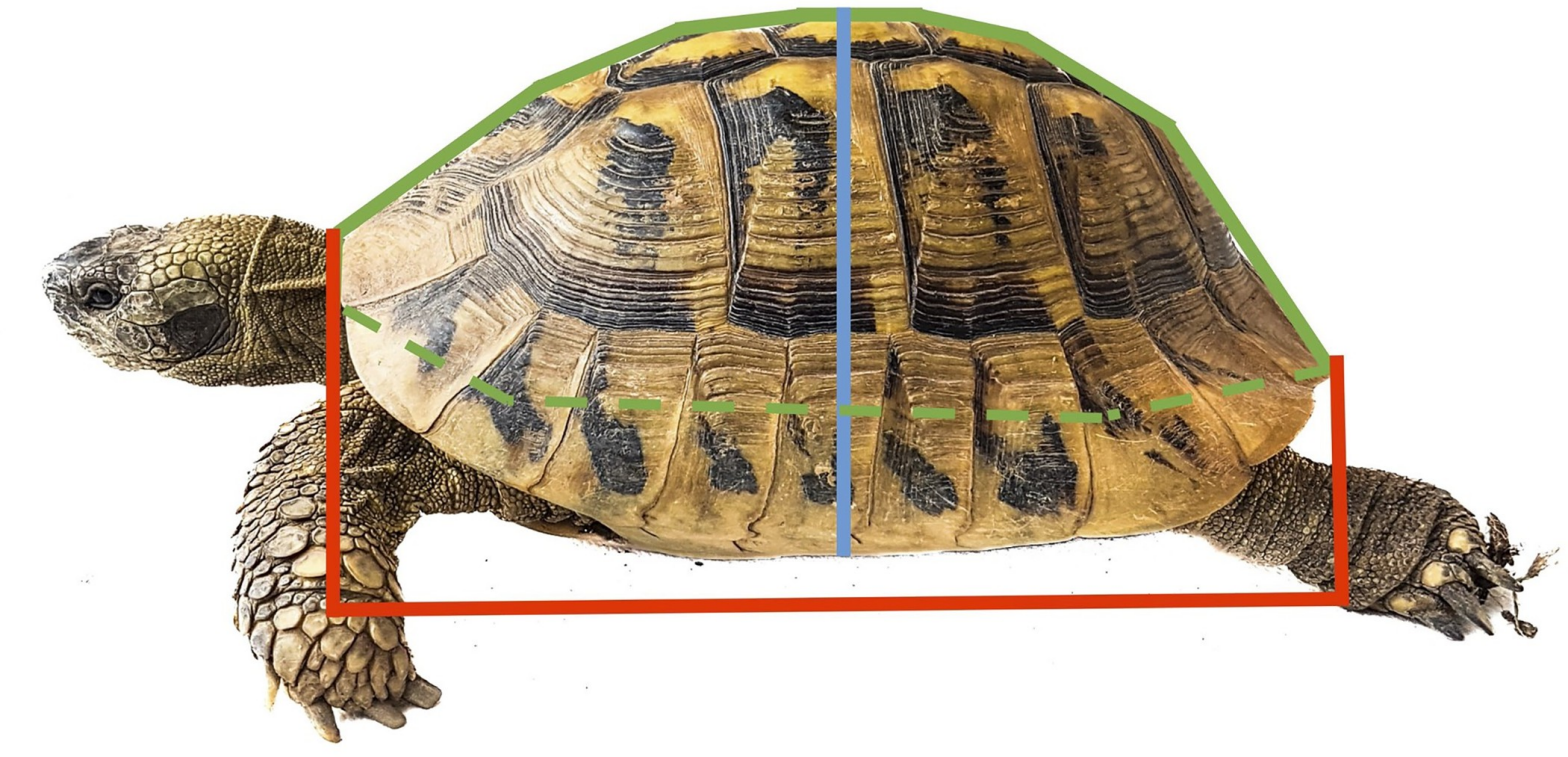
Hermann’s Tortoise with marked measurement points. Straight carapace length (from nuchal notch to caudal notch) demonstrated with the red line, carapace height and width (at the level of the third vertebral scute) demonstrated with the blue line and curved carapace and plastron circumference demonstrated with the green line.

The following parameters were then measured with an analogue DIN-862-certified 300mm caliper (Germany) to the nearest 1mm: straight carapace length (from nuchal notch to supracaudal notch), straight carapace width (at the level of the third vertebral scute) and straight carapace height (from the plastron to the highest point of the third vertebral scute; in deformed animals to the highest point). Afterwards, the circumference of the animal was measured with a flexible tape (Prym, Germany) to the nearest 1mm as follows: rounded carapace length (circumference over the length of the animal, over nuchal notch and supracaudal notch) and rounded carapace width (circumference over the width of the carapace at the level of the third vertebral scute, in deformed animals to the highest point) (Fig 1).

The degree of pyramidal growth was determined according to grades given by Wiesner and Iben [2, 58], to test the hypothesis that an abnormal carapace shape may affect the body mass and thus the correlation of a BCS and a mathematic equation. At the time of data collection, the degree of pyramiding was noted as follows: 0 = no pyramiding, 1 = slight pyramiding, 2 = moderate pyramiding, and 3 = high level of pyramiding. Furthermore, the shape of the carapace was considered as follows: 0 = normal shape; 1 = slightly deformed (e.g., slightly flattened); 2 = moderately deformed; 3 = highly deformed (e.g., distinctive hat shape). To facilitate statistical analysis, the scores were pooled as follows: “pyramiding” (degrees 2–4) or “no pyramiding” (degrees 0–1), “deformation” (degrees 2–4) or “no deformation” (degrees 0–1). The assessment of deformity was inspired by the grades of carapace deformation by Bauer et al. [2].

### Body condition scoring

Following the measurement of the carapace, the body condition score was assessed. Prior starting the study, two trained veterinarians evaluated tortoises to identify key skeletal elements for body condition assessment in Hermann’s Tortoises based on a published body condition score for Mojave Desert Tortoises (*Gopherus agassizii)* [48]. The body condition score was then modified into a five-point scale and used for assessment in the present study (Table 3).

**Table 3:** Body condition scoring sheet for Hermann’s Tortoises (*Testudo hermanni*) used in the present study.

BCS					
	1/5 (emaciated)	2/5 (underweight)	3/5 (good)	4/5 (overweight)	5/5 (obese)
**Vertebral spine (neck fully outstretched)**	very prominent and sharp, no muscle layer palpable, deep withdrawal into shell possible	prominent, easily palpable, sparse muscle layer palpable	palpable on light pressure, moderate amount of muscle mass	palpable only on pressure, muscle mass prominent	not palpable, tissue very prominent, extruding from shell
**Distal Forelimbs**	muscle atrophy, may appear concave, bones easily palpable	slight atrophy, muscles appear straight, bones palpable	well-developed musculature, limbs appear convex, bones are not palpable	muscle mass convex, very prominent	muscle mass very prominent, appears rounded
**Dorsal head shape**	concave, atrophy of temporalis muscle, Crista sagittalis visible, eyes sunken	straight appearance	straight to bi-lobe appearance	bi-lobe appearance	bi-lobe to rounded appearance
**Fat depots palpable**	no	no	no	slight	yes

**Fig 2:**
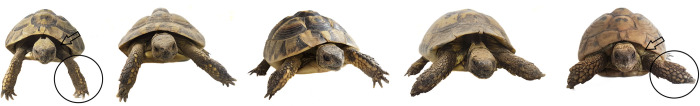
Adult Hermann’s Tortoises with different body condition scores (BCS). From left to right: BCS 2.0, BCS 2.5, BCS 3.0, BCS 4.0, BCS 4.5. Notice the deep withdrawal into the shell (arrow) and the muscle atrophy in the forelimbs (circle) of the tortoise in poor condition (BCS 2.0), compared to the tissue bulk around the neck and shoulders (arrow) and prominent muscle mass in the forelimbs (circle) of the tortoise in high condition (BCS 4.5).

**Fig 3:**
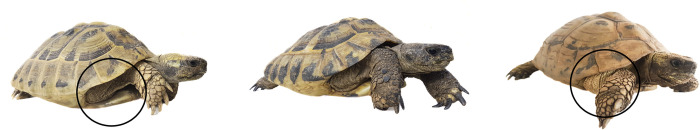
Adult Hermann’s Tortoises with different body condition scores (BCS). Tortoises in poor (left, BCS 2.0), good (center, BCS 3.0) and high (right, BCS 4.5) condition showing the difference in tissue bulk around the axillar region between these condition categories (circle).

First, each individual animal was visually examined by its head shape, limb shape, and whether it was deeply drawn into the shell due to a lack of tissue mass or rather bulging out (Figs 2 and 3). Afterwards, the animal was palpated manually. A score between one and five was then assigned based on the average score, whereby the evaluation of the vertebral spine and distal forelimbs carried more weight than the head shape and fat depot palpability. Intermediate steps were possible for animals that did not fit into one category.

Finally, using the measurements described above, the following equations were calculated in the Microsoft Excel application for each individual: Jackson’s ratio [18, 19], body condition index [20], volume condition index of Nagy [24], volume condition index of Loehr [25], circumferential product using rounded carapace measurements (Table 1). As suggested by Willemsen et al. [14] different equations were used for evaluation of the body condition index in the subspecies *T*. *h*. *boettgeri* and *T*. *h*. *hermanni* respectively.

The complete data set of Hermann’s tortoises measured in the present study can be found in the supporting information (S1 File).

### Permits and ethics statement

The ethics committee of the veterinary department of the LMU Munich has approved the research (AZ 224-09-07-2020). The application and approval letter can be found in the supporting information (S2 File). In Germany, since all tortoises were privately owned, the approval for the field site access had been given by the owners. In France, this project was conducted under the permits delivered by prefectural authorities (Departmental Direction of Territory and Sea in the Département du Var) on July 1, 2021, and February 26, 2013 (Cerfa N° 13 616*01). Participant consent was informed prior to the study, given verbally, and witnessed by PD Dr. Petra Kölle. If no consent was given, the tortoise owner was not visited. No minors were included in the study. The animal data were recorded anonymously directly on site.

### Statistical analysis

Statistics were performed using R Statistical Software version 4.3.1 (2023-06-16).

To visualize the data and study correlations between parameters, the non-parametric Spearman correlation matrix was used. This allowed testing whether the body condition score correlated with pre-existing systems for measuring body condition, which relationship was strongest and whether the measurement systems were interchangeable. In each correlation matrix, the data were first checked for linearity of correlation and normality of distribution.

The hypothesis was first tested in the entire population, taking into account all animals with the exception of those in which individual parameters were missing. In such a case, only the correlations that were possible were performed on this animal (i.e., only correlation of BCS and body condition index). The data was then subdivided according to the parameters of subspecies, origin, husbandry, and sex to detect influencing factors. Additionally, it was assessed whether there was an influence of deformation and pyramiding in the correlation between the BCS and the measurement method with the strongest relationship in the total population, which in this case were the volume indices.

The correlation coefficient rho in principal ranges from -1 to +1, whereas 0 indicates that there is no linear or monotonic association between two variables, and rho +1 or -1 signifies the strongest possible relationship. The translation of the correlation coefficient rho into terms such as "weak", "moderate" or "strong" correlation is inconsistent in literature. Instead of clear cut-off values, it was suggested that rather an interpretation be made in the context of the question posed [59].

Furthermore, it was investigated whether there was sexual dimorphism and a difference in size between the French and German animals in the data set, hypothesizing that female specimens from Germany (mostly *T*. *h*. *boettgeri*) were presumably larger and heavier than their male or French (*T*. *h*. *hermanni*) counterparts. To this end, the two samples t-test and Mann-Whitney U test were used. First, data were checked for normality of distribution by using Shapiro-Wilk normality tests. When data were not normally distributed, as indicated by low Shapiro-Wilk p-values (p < 0.05) in at least one of the groups, Mann-Whitney U test was used. When data were normally distributed, as indicated by high Shapiro-Wilk p-values (p > 0.05) in both samples, the homogeneity of variances was checked with Levene’s Test for Homogeneity of Variance. When variances between two samples were similar (p > 0.05), Student’s-t-test was used. When variances were different (p < 0.05), Welch’s t-test was used.

Level of statistical significance was set at p < 0.05.

## Results

### Correlation between body condition score and shell measurements

In the total population, there was a significant positive correlation (p <0.05) between the body condition score and all measurement systems. The strength of the correlation differed slightly when considering the subspecies *T*. *h*. *boettgeri* and *T*. *h*. *hermanni* (Table 4).

**Table 4:** Strength of correlation between BCS and measurement methods ranging from strongest to weakest (from left to right).

**Total population**				
	**Volume indices**	**Circumferential product**	**BCI**	**Jackson’s ratio**
**BCS**	0.373***^a^	0.367***	0.319***	0.286***
*T*. *h*. *boettgeri*				
	**Volume indices**	**BCI**	**Circumferential product**	**Jackson’s ratio**
**BCS**	0.405***	0.336***	0.334***	0.316***
*T*. *h*. *hermanni*				
	**Circumferential product**	**Volume indices**	**BCI**	**Jackson’s ratio**
**BCS**	0.482***	0.340***	0.288**	0.215*

^a^Strength of correlation is indicated by the Spearman correlation coefficient rho. Stars indicate p-values

* = p < 0.05

** = p < 0.01

*** = p < 0.001

**Fig 4:**
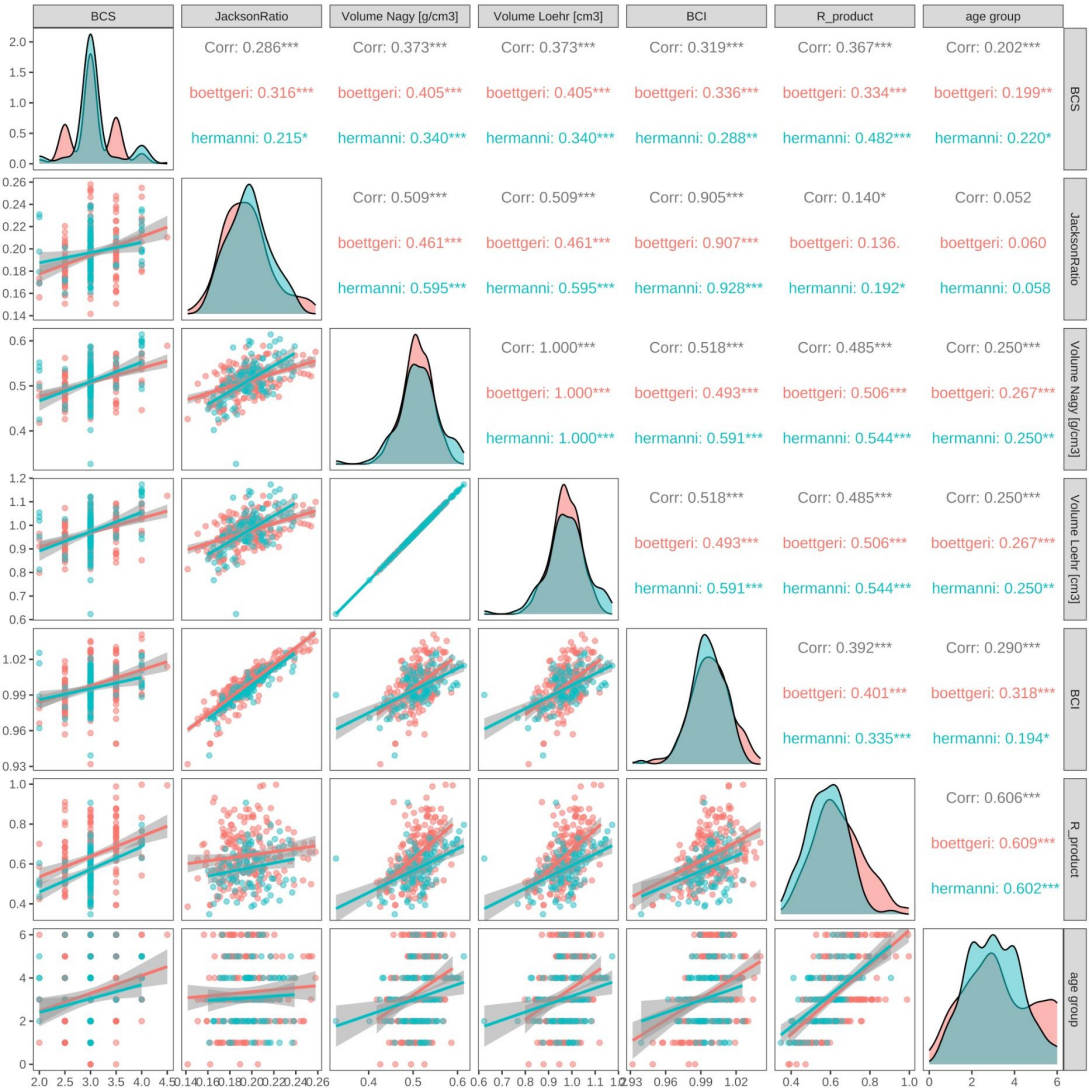
Data visualization in the spearman correlation matrix of the total population. Spearman correlation coefficient rho is indicated in gray (total population), pink (*Testudo hermanni boettgeri*) and blue numbers (*Testudo hermanni* hermanni), p-values are indicated by stars: * = p < 0.05, ** = p < 0.01, *** = p < 0.001.

The full correlation matrix, including the comparison between measurement methods, is shown in Fig 4.

To determine the influence of the origin of the animals, the data set was split up and the same correlations as shown above were conducted with German and French animals. In the total German population, the correlation of BCS and the volume indices showed the strongest relationship (rho: 0.387, p<0.001), while in the total French population, it was the correlation of BCS with the circumferential product (rho: 0.543, p<0.001). In both cases, the correlation of BCS and Jackson’s ratio showed the lowest relationship (German tortoises: rho: 0.304, p<0.001, French tortoises: rho: 0.221, p<0.05).

Further differences emerged when the data was broken down by sex. In German tortoises, there was an overall significant positive correlation of the BCS and all measurement methods in male and female specimens. In females, the BCS to volume indices correlation showed the highest effect size (rho: 0.420, p<0.001), and the BCS to Jackson’s Ratio the lowest effect size (rho: 0.324, p<0.001). In males, the BCS to BCI correlation showed the highest effect size (rho: 0.351, p<0.001), while the BCS to volume indices correlation showed the lowest effect size (rho: 0.266, p<0.01). While in French tortoises, all correlations were significant in females, with the BCS to circumferential product correlation showing the highest effect size (rho: 0.627, p<0.001) and the BCS to Jackson’s ratio correlation showing the lowest effect size (rho: 0.290, p<0.05), in male tortoises there was no significant correlation of the BCS with any equation.

The most surprising discovery was made when checking the data for an influence of the husbandry of the animals. While in captive animals, there was a significant correlation between the body condition score and the volume indices (rho: 0.645, p<0.001), the circumferential product (rho: 0.870, p<0.001) and the BCI (rho: 0.419, p<0.05), but not for the Jackson’s ratio, astonishingly, in free-ranging animals, there was no significant correlation between the body condition score and any other measurement method at all.

Regarding the effect of carapace deformation and pyramidal growth, a correlation was performed between the body condition score and the measurement method with the strongest correlation in the total population, which in the case of the current study was the volume index by Loehr et al. [25]. In the data set, animals without carapace deformation or pyramidal growth showed a significant positive correlation (rho: 0.372, p<0.001). The number of deformed animals was however too small to draw statistical evidence.

### Size differences and sexual dimorphism

**Fig 5:**
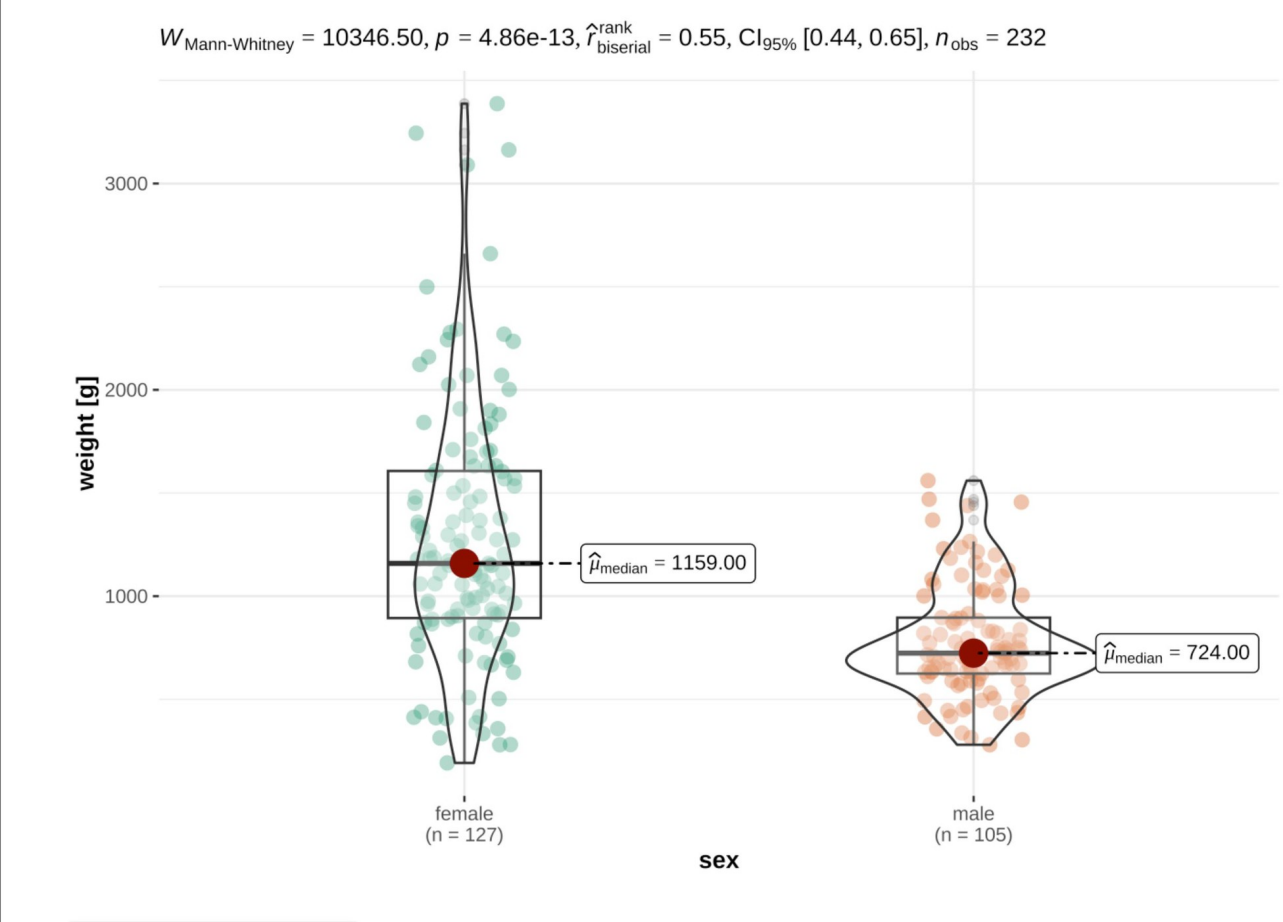
Violin plots of variance distribution of weight in male and female German tortoises. Individual data points are distributed as colored dots within each category, with the box indicating the middle half of the data set and the width of the box expanding with the number of data points. The median values are shown as red dots.

**Fig 6:**
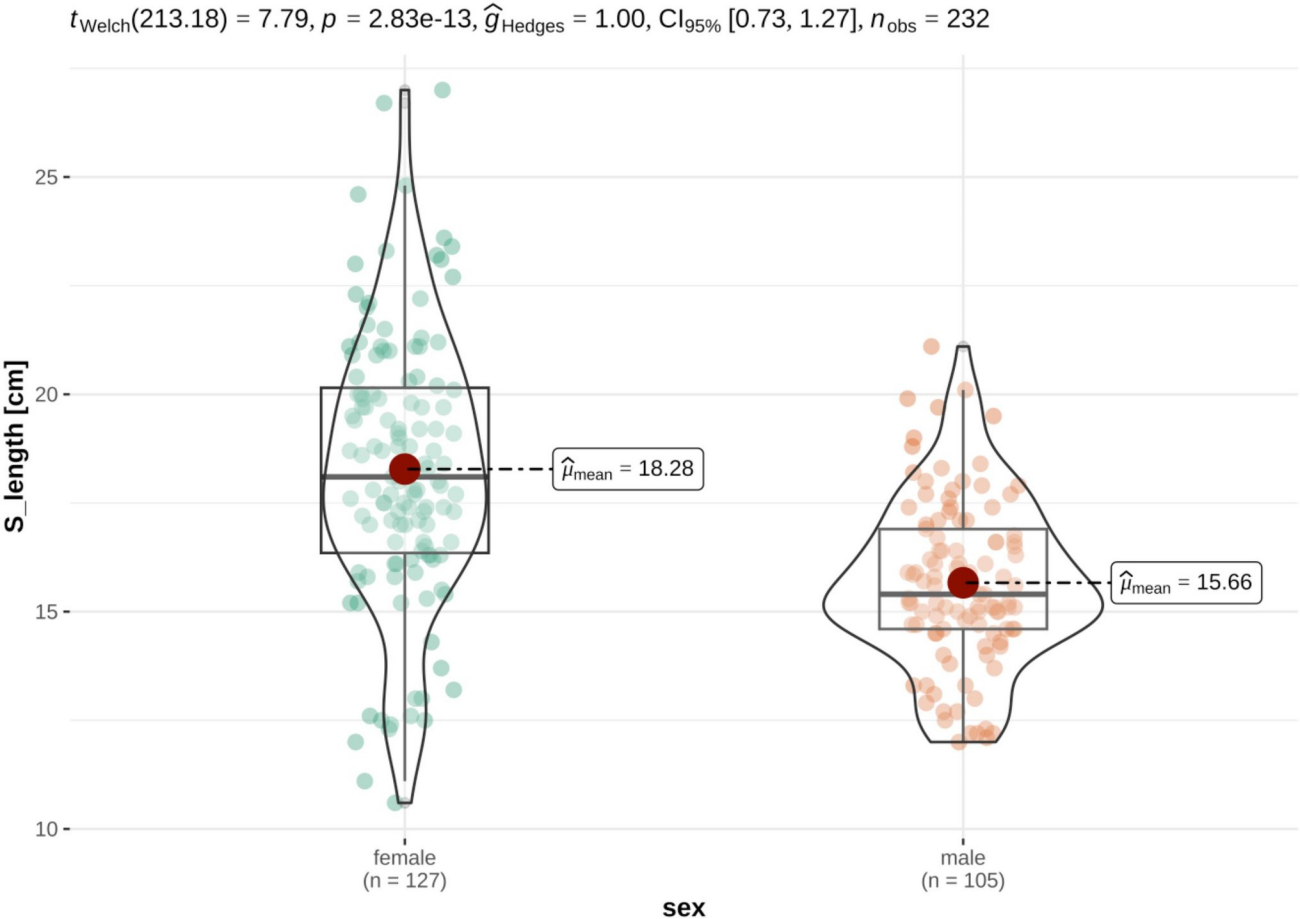
Violin plots of variance distribution of carapace length in male and female German tortoises. Individual data points are distributed as colored dots within each category, with the box indicating the middle half of the data set and the width of the box expanding with the number of data points. The mean values are shown as red dots. This effect was also seen in French tortoises, where female tortoises were significantly heavier (females: mean 816.53 ± 192.24g, males: mean 423.69 ± 111.08g) longer (females: median 15,96 ± 1,57cm males: median 13.05 ± 1.51cm), broader (female: median 11.84 ± 0.95cm, males: median 10.26 ± 1.04cm) and higher (female: median 8.13 ± 0.59cm, males: median 6.58 ± 0.53cm) than their male counterparts (Figs 7 and 8).

For the investigations on size differences and to check for sexual dimorphism, only animals for which all measurement data was available were considered. As expected, distinct sexual dimorphism was discovered in the data set, with female German tortoises being significantly heavier and larger than their male counterparts. In detail, German tortoises were heavier (females: median 1159.00 ± 713.00g, males: median 724.00 ± 271.00g), longer (females: mean 18.28 ± 3.12cm, males: mean 15.66 ± 1.90cm), broader (female: mean 13.97 ± 2.28cm, males: mean 12.48 ± 1.50cm) and higher (female: mean 8.96 ± 1.55cm, males: mean 7.69 ± 0.90cm) and than their male counterparts (Figs 5 and 6).

**Fig 7:**
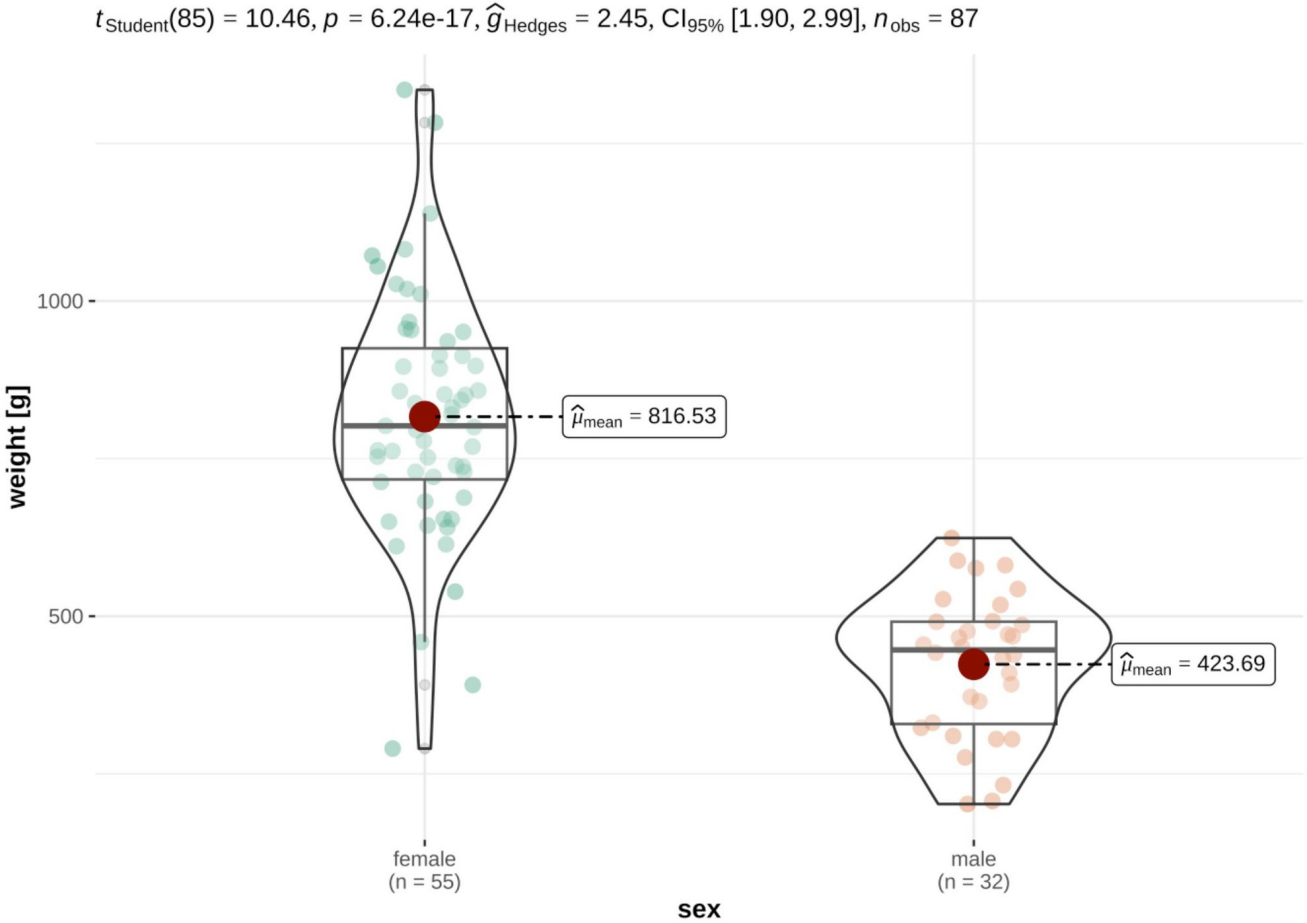
Violin plots of variance distribution of weight in male and female French tortoises. Individual data points are distributed as colored dots within each category, with the box indicating the middle half of the data set and the width of the box expanding with the number of data points. The mean values are shown as red dots.

**Fig 8:**
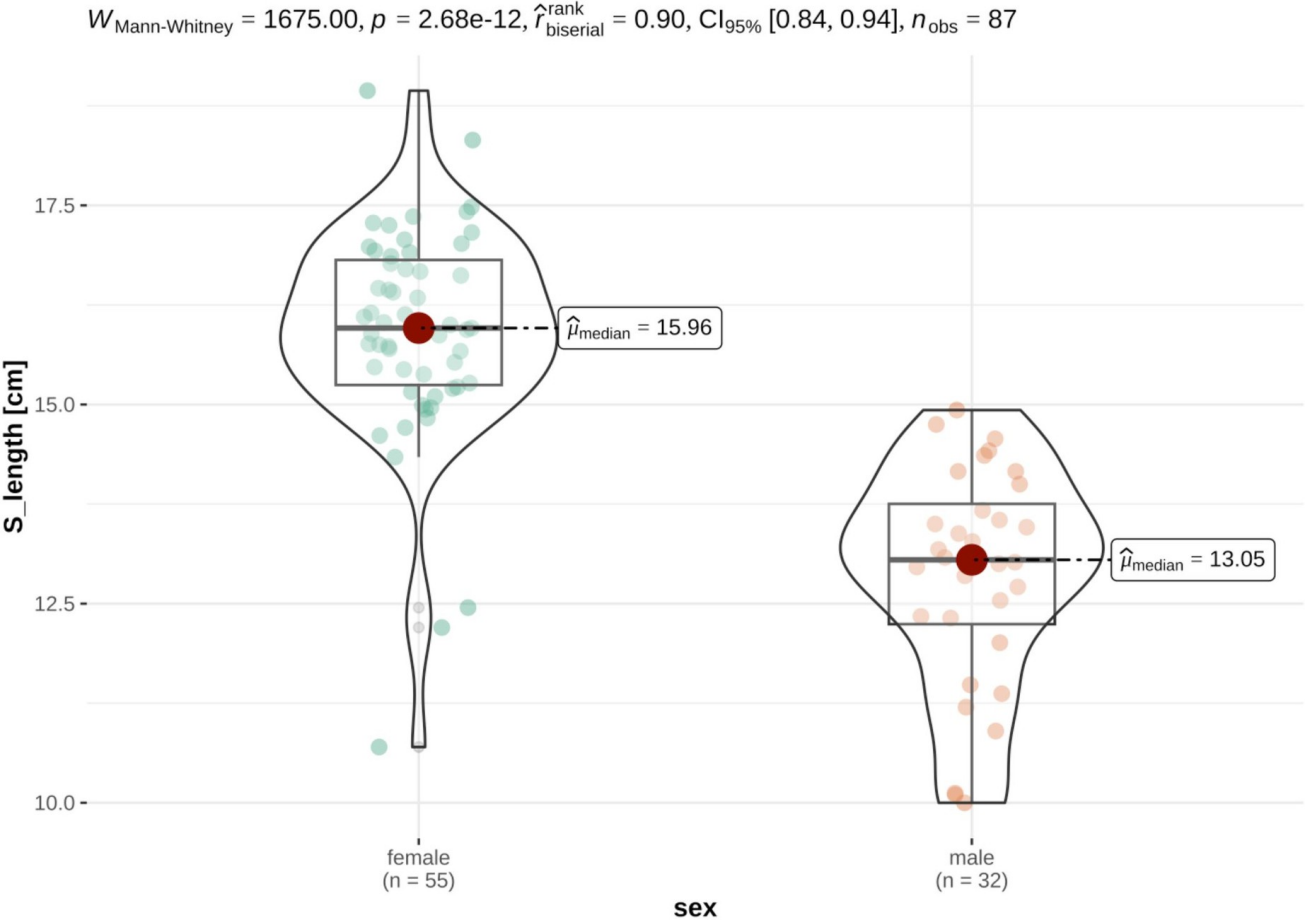
Violin plots of variance distribution of carapace length in male and female French tortoises. Individual data points are distributed as colored dots within each category, with the box indicating the middle half of the data set and the width of the box expanding with the number of data points. The median values are shown as red dots.

In total, German tortoises (including mostly *T*. *h*. *boettgeri*) were significantly heavier and larger than French tortoises (*T*. *h*. *hermanni*). This effect also persisted when including only animals with roughly ideal body condition (2.5–3.5). These animals showed a median body weight of 641.0g in French tortoises (n = 71) and 914.5g in German tortoises (n = 216). They were in median 14.9cm (French tortoises, n = 71) and 16.9cm (German tortoises, n = 216) long respectively, had a median width of 11.2cm (French tortoises, n = 71) and 13.1cm (German tortoises, n = 216) and a median height of 7.4cm (French tortoises, n = 71) and 8.3cm (German tortoises, n = 216).

A considerable proportion of animals in the data set were in normal condition (62.2%), while 21.7% were over-conditioned and 16.1% were under-conditioned. In general, female German tortoises had higher body condition scores (56.1% in good condition, 28.8% over-conditioned, 15.1% under-conditioned) than males (54.7% in good condition, 19.7% over-conditioned, 25.6% under-conditioned). The same applied to French female tortoises that displayed higher body condition scores (77.2% in good condition, 21.0% over-conditioned, 1.8% under-conditioned) compared to males (90.9% in good condition, 0.0% over-conditioned, 9.1% under-conditioned).

## Discussion

Although various techniques are known for assessing the body condition of mammals, their application in tortoises is limited. Current gold standard for measuring body composition including fat and muscle proportions in cats and dogs is the dual-energy X-ray absorptiometry [60, 61]. In principle, this tool is available also to chelonians, but the precision is constrained due to the overlay of the entire coelomic cavity with the carapace [62–64]. In addition, proper positioning of the animal is crucial to ensure accuracy in the measurement [65], therefore the animal should ideally be anesthetized. This also applies for computed tomography [66, 67]. In addition to the great expenditures and excessive costs for these procedures, most practices further lack the equipment to perform these assessments. While blood analysis is generally a valuable tool for health screening in reptiles, the determination of triglycerides has no reliable diagnostic value for the assessment of nutritional status due to the strong influence by vitellogenesis [12, 68–72]. Ultrasound, in principle, offers the possibility to measure depth of the subcutaneous fat layer, especially in larger sea turtles [73]. However, in addition to the difficulties presented with smaller individuals, reference values in tortoises do not appear to have been established and exact localization and interpretation of intracoelomic fat deposits remains challenging. Other methods for the measurement of body composition, such as total lipid extraction, gas dilution, chemical analysis, or electrical conductivity [74], are elaborate and generate considerable stress for the animals.

The purpose of the present study was to examine whether the body condition score could facilitate the assessment of body condition compared to the mathematical equations that currently represent the most viable alternative for the non-invasive assessment of tortoises. To this end, the significant positive correlation between the body condition score and the mathematic equations in captive tortoises is of particular interest to the veterinarian community. The relationship represented a medium magnitude in the data set, combined with very low p-values, suggesting a significant relationship that is not attributable to chance, but that the results should also be interpreted with consideration. The positive correlation signifies a linear association [75]. Therefore, the higher the index to predict body condition, the higher also the body condition score of an animal, and vice versa. These findings support the hypothesis that a BCS provides comparable results for body condition assessment as mathematical equations.

However, evidence was found that the mathematical equations may not always display the body condition of the tortoise correctly. Regardless of the overall correlation of the measurement systems with the BCS, individual animals with normal or high values in the carapace measurements at the same time displayed a body condition score that was significantly too low and suggested a necessity for medical attention. Conversely, a high measurement score could have led to the false assumption that the animal was in good condition. Pathological reasons for increased weight with low body condition include stasis of the bladder, lithophagy, oedema, egg peritonitis or liver disease [21–23]. These findings are in accordance with Jacobson [21], who argued on many factors limiting body condition assessment based on measurements only: Firstly, when only considering the carapace length and the weight of the animal, sexual dimorphism in tortoises is ignored. The differently shaped shell of female specimens may however be heavier than the male counterpart and therefore wrongly suggest a higher body condition [22]. The quality of bone including that of the shell may also play a part in body mass. Another issue to consider is the influence of hydration and bladder filling, which can account for up to 30% of the body weight of some tortoises [24].

Concurrently, individual animals with low values in the mathematical equations exhibited high body condition scores at the same time. Considerations of Willemsen and Hailey offer a possible answer to this phenomenon: since their volume is mostly constrained within their shell, fat might increase at the expense of higher density tissue such as muscle and thus lower the body mass of a tortoise with increasing condition [20]. However, intrusive studies would be required to confirm such a theory. Although the body condition score cannot be the sole predictor of health status, this discrepancy underlines the importance of clinical assessment of each animal.

Further data collection on animals with extremely low (<2.0) or extremely high body condition score (>4.5) values might add to determine further influences, which are missing in the data set of the current study. It must be considered that the survey was based on the voluntary participation of tortoise keepers. Participation is expected to be higher among pet owners who keep their animals in a species-appropriate manner than among owners whose animals correspond to the extremes. Further effort was made with an e-mail survey to reptile veterinarians, but there was little feedback. Nevertheless, the BCS range of 2.0 to 4.5 in captive tortoises appeared sufficient to demonstrate the correlation pattern for the BCS.

Despite the above-mentioned points, calculations cause only minimal disturbance to the animal and can be repeatedly performed with little training. They may therefore provide a good basis for assessment especially for shy or free-ranging animals that are contracted back into their shell, and may due to their objectivity also be used retrospectively, which is of great interest for ecological research.

Interestingly, the findings of the present study contrast with previous results by Gimmel et al. [49], who observed no correlation between a body condition score and a body condition index or the Jackson’s ratio in Hermann’s Tortoises. One hypothesis is that the larger sample size in the current study could have led to different results. This could be particularly true concerning the sometimes strong individual variation that was observed in the present study and which could impair the statistical significance if the sample size is too low. Furthermore, a different body condition scoring sheet was used in their study, limiting the assessment points to the palpation of cervical and tail vertebrae. The exact background is difficult to fathom, as only an abstract of this study has been published to date and no information on the anamnesis of subspecies, age or carapace deformation can be given so far.

As expected, the strongest correlations of the BCS in the total population were observed with the volume indices, the lowest with the Jackson’s ratio. The volume indices with their additional carapace measurement points provide more data and should allow more accurate growth-independent predictions, while the Jackson’s ratio may be biased due to factors mentioned further above.

Concerning the two subspecies of *T*. *hermanni*, there was a significant difference in the absolute size of the animals, with *T*. *h*. *boettgeri* being larger than *T*. *h*. *hermanni*. This result is consistent with previous literature [55, 76]. In the data set of the present study, there were only minor differences in the strength of the correlations in the total population.

Contrary to these findings in the total population, it is interesting to note the lack of correlation of any methods in male French tortoises. Furthermore, the strength of each correlation differed between German and French tortoises. While the underlying factors are not yet fully understood, the apparent lack of correlation can be attributed to several reasons. The use of a BCS, like any subjective assessment in veterinary practice, requires a certain amount of training to achieve the best results. Since the data were collected by different individuals, some variance due to the subjectivity of the assessors cannot be ruled out, even though the most detailed assistance possible was provided by guidelines including sample photographs beforehand and all examiners were used to handling and assessing tortoises frequently. While this subjectivity lies in the nature of the BCS [37], it can be improved through repeated use and training. Lagerström [27] also noted some discrepancies in body condition scoring results when assessed by different examiners; however, the average scores appeared to be consistent. It therefore remains questionable whether such a large effect as seen in the results of the present study can be caused by examiner variance alone. Repeated data collection on the same animals by different examiners would be needed to determine the magnitude of this effect precisely. A possible source of error could also lie in the narrow BCS range of the French tortoises, as more than 90% of male tortoises had a BCS of 3.0, which limited the sample size for over-conditioned and under-conditioned animals in contrast to the German tortoises, where only 54% of males showed a BCS of 3.0. In addition, the more dome-shaped carapace of the French subspecies *T*. *h*. *hermanni* might have influenced calculations differently. For the most accurate measurement, an individual approach might be required, and further underlying factors in body condition assessment may yet need to be identified.

This may also apply to free-ranging animals, which displayed no correlation of the body condition score to any other measurement system. It is discussed that data from free-ranging tortoises may not always be directly applicable to captive tortoises [14], however, precise studies on the background and influencing matter of this nature are lacking. In addition to the reasons mentioned above, seasonal differences or regional variations may have a significant impact [12, 14].

Certain differences arise from the varying climate zones. Germany has a cool temperate climate, while the Mediterranean climate is warm and mild. These seasonal patterns, which result in differences in heat, rainfall, and subsequently food availability, are expected to lead to different growth patterns, as observed in other populations [14]. Furthermore, it is assumed that microclimate factors, such as humidity and day-night subsidence, have an effect on shell shape, particularly in the development of pyramidal growth syndrome [2, 58].

Phenotypic plasticity is a common occurrence in *T*. *hermanni*, reflecting the species’ ability to adapt to various habitats throughout its range. Several studies have described differences in body size, shell shape and morphology among populations [57, 77–81]. These variations are believed to be influenced by factors such as survival rate, growth rate, mortality rate, genetics, and response to environmental conditions [80]. It is suggested that a north-south cline may also play a role. In this context, Bergmann’s rule suggests that animals in colder climates tend to have larger body sizes, which helps them retain heat due to their reduced surface area [82]. Studies on Italian and Greek animals partially support this hypothesis [81, 83], although Willemsen and Hailey [81] suggest that differences in adult mortality, rather than thermo-regulation, may be the ultimate cause of size variation. In a recent study conducted by Duro et al. [80], this phenomenon was observed exlusively in females. Duro et al. [80] argue that the larger body size may be an adaptation to a shorter breeding season in this region, resulting in increased reproductive success through larger clutches.

Geographical barriers and dramatic climate changes during Pleistocene and Pliocene are widely believed to have contributed to the particularly strong biological diversification of the subspecies *T*. *h*. *boettgeri* in several regufia in the Balkans [55, 77]. There has been repeated discussion about a third subspecies, *Testudo hercegovinensis* (*Testudo graeca var*. *hercegovinensis*) [84]. Although this assumption has been rejected by genetic analyses conducted by Fritz et al. [55], it highlights the potential diversity within the species. Đurakić and Milankov recently suggested that reassessment of a subspecific ranking within *T*. *h*. *boettgeri* may be necessary [79]. Geographical barriers can also result in reduced genetic variability, which may lead to skeletal anomalies due to inbreeding [78]. Furthermore, Soler et al. [85] have extensively discussed the issue of genetic pollution resulting from intraspecific cross-breeding, which is considered a significant threat to the genetic diversity of *T*. *hermanni*.

Hermann’s Tortoises exhibit pronounced sexual dimorphism, as confirmed by the findings of the present study. While ecological differences and natural selection also contribute to the degree of sexual dimorphism, general shell shape is likely influenced by fecundity selection in females (dome shape to carry more eggs) and sexual selection in males (stability and support during mating) [56, 57, 86, 87].

Due to the wide distribution area of *T*. *hermanni*, morphological variation can be considerable and the results of the study may not be applicable to all populations. One further hypothesis is that the sample size was too low to provide statistical evidence, as might be the case for the lack of correlation in the study by Gimmel et al [49], though this can only be speculated upon without further studies.

While these results suggest that the BCS can be applied to captive female *T*. *h*. *hermanni* as well as captive *T*. *h*. *boettgeri* of either sex, caution should be advised when applying the score to male *T*. *h*. *hermanni* or free-ranging animals without prior confirming studies.

The significant correlation between the age group of the animal and the body condition score, volume indices, BCI and circumferential product should be interpreted separately. As logical consequence of growth, the circumferential product of a tortoise increases with age. Contrary to expectations, however, body condition scores and the volume indices increased with age in the data set, thus the older the animals were, the higher their body condition was. In detail, the youngest animals in the study (up until 5 years of age) predominantly showed a good body condition score on average (3.05), while animals of 5–10 years had the lowest scores (2.90), which then increased further the older the animals became (BCS average score of 3.23 in ages up to 60 years). One hypothesis for this observation is that young tortoises tend to allocate excess energy to faster growth rather than fat storage, making it unlikely to find young animals with higher body condition score [28, 88]. Also, emaciated young animals in the wild are quickly endangered by predators, making it unlikely to find these animals for data measurement. Many factors play a role in the growth of tortoises, not all of which are yet fully understood. Sarcopenia is evident in older mammals [89]. Whether this also plays a part in tortoises has been proposed [5] but not yet sufficiently researched, given the enormous lifespan of tortoises and the considerable effort involved in recording body condition data of individuals throughout their entire lives. It is conceivable that an older tortoise has certain advantages in food and territorial selection due to its size compared to a younger conspecific, comparable to such findings in snakes [90].

In this regard, it should be emphasized that, while being constructed on a similar base model, the principle of the body condition score in the present study is not identical to that of domestic mammals. As mentioned before, the sole measurement of subcutaneous fat and protrusion of bony structures, as considered in other animals, does not seem feasible for tortoises with their different skeletal anatomy and fat distribution. Furthermore, the body condition of an animal is not limited to fat volume alone but also reflects muscle content and hydration status. The body condition score was therefore determined also by the development of the musculature, as is considered in a separate muscle condition score (MCS) in dogs and cats [91, 92].

The difference in weight and carapace measurements between captive and free-ranging animals confirms previous research [28, 88] and may be based on several reasons. For one, the life expectancy of captive tortoises is presumed to expand with healthcare, analogue to other species kept in human captivity. As such, many of the measured captive animals were 60 years and older, while only one free-ranging animal was older than 30 years of age. Furthermore, in the natural range of Hermann’s Tortoises, animals will spend a considerable proportion of their daily activity searching for appropriate food [11, 12, 93]. In contrast, captive tortoises are frequently fed at least once or several times a day, often ad libitum and with a comprehensive selection of wild herbs, salads, and vegetables. In addition, even a large outdoor enclosure provides fewer opportunities for physical activities than natural habitats. The captive tortoises therefore must expend considerably less energy in their search for food, mates, and egg-laying sites. An energy surplus and consequently faster growth is fostered by humans compared to nature [28, 88, 94, 95].

The present study aimed to supplement and facilitate general health examination in Hermann’s Tortoises. With evidence of the data set, it is recommended not to rely on either the mathematical equations or the body condition score only. Clinical evaluation of additional health markers, in-depth medical history regarding feeding and husbandry regime or blood evaluation of organ status should be considered as a part of good veterinary care.

## Conclusion

The focus of the present study was on the practicability of a non-invasive, simple, and low-cost tool for assessing nutritional status and body condition of Hermann’s Tortoises. Due to anatomical features and the physiologically different distribution of fat tissue, it is not possible to directly transfer the body condition scoring system from domestic mammals to tortoises. Instead, the application of a tortoise specific body condition score inlcuding both fat and muscle distribution is proposed for condition assessment. The application of a body condition score is a subjective method and requires training to achieve the optimal result. While most of the results of the present study were anticipated, such as sexual dimorphism or size differences between the subspecies *Testudo hermanni hermanni* and *Testudo hermanni boettgeri*, not all results were consistent with previous studies, stressing the importance of further investigating the magnitude of influencing factors on body condition in tortoises. In free-ranging Hermann’s Tortoises, further studies need to be conducted to confirm or disprove the use of a BCS. However, the findings of the present study support the hypothesis that the body condition evaluation based on a BCS leads to comparable results as an assessment derived from mathematical equations in captive Hermann’s Tortoises. While shell measurement systems may provide initial information especially in shy or free-ranging animals, they can fail to recognize low or high body condition. Therefore, despite their objectivity, clinical evaluation of the tortoise, including a body condition score, remains mandatory for health care in tortoises.

## Supporting information

S1 File Complete raw data set of Hermann’s tortoises measured in the present study. (PDF)

S2 File Application and approval letter by the ethics committee of the veterinary faculty of LMU Munich. (PDF)
