# Supplementary material for: Body condition scoring facilitates healthcare monitoring in Hermann’s Tortoises (Testudo hermanni ssp.)
Source: PLoS One. 2024 Apr 18;19(4):e0301892. doi: 10.1371/journal.pone.0301892 (PMC11025769; doi:10.1371/journal.pone.0301892)
Supplement: S1 File — (PDF) [file pone.0301892.s001.pdf]

ID	Origin	sex	BCS	weight [g]	S_length [cm]	S_length_BCI [mm]	S_width [cm]	S_height [cm]	R_width [cm]	R_length [cm]	pyramiding	deformation	JacksonRatio	Volume Nagy [g/cm3]	Volume Loehr [cm3]	BCI	R_product	Species	age group	husbandry
015	German	male	2,0	259	11,1	111	8,4	5,1	23,9		no pyramiding	no deformation	0,19	0,54	1,04	0,97		boettgeri	1	wild
148	German	female	3,0	186	10,0	100	7,6	5,1	20,4		no pyramiding	no deformation	0,19	0,48	0,92	0,96		boettgeri	1	captive
179	German	female	3,5	300	10,9	109	8,8	5,8	24,1		no pyramiding	no deformation	0,23	0,54	1,03	1,01		boettgeri	1	captive
180	German	female	3,0	179	10,0	100	7,9	5,3	21,5		no pyramiding	no deformation	0,18	0,43	0,82	0,96		boettgeri	1	captive
181	German	female	3,0	221	10,2	102	8,0	5,3	21,7		no pyramiding	no deformation	0,21	0,51	0,98	0,98		boettgeri	1	captive
183	German	female	3,0	299	11,0	110	8,5	5,9	24,0		no pyramiding	no deformation	0,22	0,54	1,04	1,00		boettgeri	1	captive
184	German	female	3,0	242	10,5	105	8,5	5,4	23,2		no pyramiding	no deformation	0,21	0,50	0,96	0,99		boettgeri	1	captive
185	German	female	3,0	270	11,0	110	8,3	5,8	23,0		no pyramiding	no deformation	0,20	0,51	0,97	0,98		boettgeri	1	captive
204	German	female	3,0	302	11,3	113	9,0	5,3	23,9		no pyramiding	no deformation	0,21	0,56	1,07	0,99		boettgeri	1	captive
281	German	male	3,0	278	11,3	113	8,8	5,3	23,2		no pyramiding	no deformation	0,19	0,53	1,01	0,98		boettgeri	1	captive
369	German	male	3,0	246	11,2	112	8,6	5,2	22,4		pyramiding	no deformation	0,18	0,49	0,94	0,96		boettgeri	0	captive
373	German	male	3,0	179	10,0	100	8,0	4,9	20,7		pyramiding	no deformation	0,18	0,46	0,87	0,96		boettgeri	0	captive
374	German	male	3,0	162	11,0	110	8,0	4,8	20,5		no pyramiding	no deformation	0,12	0,38	0,73	0,89		boettgeri	0	captive
382	German	female	3,0	252	10,2	103	8,3	5,1	21,9		no pyramiding	no deformation	0,24	0,58	1,11	1,00		boettgeri	1	captive
500	German	male	3,0	1032	17,1	171	13,3	8,9	36,8	44,3	no pyramiding	no deformation	0,21	0,51	0,97	1,01	0,63	boettgeri	6	captive
501	German	male	3,0	1035	17,4	174	13,1	8,8	36,4	43,5	no pyramiding	no deformation	0,20	0,52	0,99	1,01	0,65	boettgeri	6	captive
502	German	male	2,5	1184	17,8	178	14,5	9,7	38,5	44,9	pyramiding	no deformation	0,21	0,47	0,90	1,02	0,68	boettgeri	6	captive
503	German	male	2,5	1020	18,0	180	13,7	9,2	36,4	45,2	pyramiding	no deformation	0,17	0,45	0,86	0,99	0,62	boettgeri	4	captive
504	German	female	2,5	3244	27,0	270	19,8	13,3	54,0	66,0	no pyramiding	no deformation	0,16	0,46	0,87	0,99	0,91	boettgeri	6	captive
505	German	female	2,5	2499	23,0	230	17,7	12,5	48,5	57,8	no pyramiding	no deformation	0,21	0,49	0,94	1,01	0,89	boettgeri	6	captive
506	German	female	2,5	1110	17,8	178	14,1	8,7	38,2	57,5	no pyramiding	no deformation	0,20	0,51	0,97	1,00	0,51	boettgeri	2	captive
507	German	male	2,5	450	13,3	133	10,5	7,3	28,8	33,8	pyramiding	no deformation	0,19	0,44	0,84	0,99	0,46	boettgeri	3	captive
508	German	female	3,0	1150	17,7	177	13,3	9,2	36,6	44,2	no pyramiding	no deformation	0,21	0,53	1,01	1,01	0,71	boettgeri	6	captive
542	German	male	2,5	663	15,1	151	12,1	7,7	31,6	37,8	pyramiding	no deformation	0,19	0,47	0,90	1,00	0,56	boettgeri	1	captive
545	German	male	2,0	314	12,3	123	9,1	5,7	24,2	30,6	pyramiding	no deformation	0,17	0,49	0,94	0,97	0,42	boettgeri	1	captive
546	German	female	3,0	711	15,4	154	10,1	7,8	31,0	38,4	pyramiding	no deformation	0,19	0,59	1,12	0,99	0,60	boettgeri	1	captive
547	German	female	2,5	313	12,3	123	8,8	5,5	23,6	30,2	pyramiding	no deformation	0,17	0,53	1,00	0,96	0,44	boettgeri	1	captive
550	German	male	3,0	337	12,1	121	9,2	6,0	25,2	31,3	pyramiding	no deformation	0,19	0,50	0,96	0,99	0,43	boettgeri	1	captive
585	German	male	2,5	1030	18,2	182	13,1	8,8	35,7	45,4	no pyramiding	no deformation	0,17	0,49	0,94	0,99	0,64	boettgeri	4	captive
586	German	female	3,0	1126	19,2	192	14,0	9,0	37,8	47,4	pyramiding	no deformation	0,16	0,47	0,89	0,97	0,63	boettgeri	2	captive
587	German	female	3,0	1587	19,2	192	15,7	9,4	40,9	48,0	no pyramiding	no deformation	0,22	0,56	1,07	1,02	0,81	boettgeri	3	captive
588	German	female	3,0	1458	19,7	197	14,3	9,6	39,6	48,8	pyramiding	no deformation	0,19	0,54	1,03	1,00	0,75	boettgeri	2	captive
589	German	female	3,0	1359	19,2	192	15,2	8,7	39,3	47,3	pyramiding	no deformation	0,19	0,54	1,02	1,00	0,73	boettgeri	3	captive
590	German	female	3,0	1059	17,8	178	13,9	9,2	37,0	44,6	pyramiding	no deformation	0,19	0,47	0,89	0,99	0,64	boettgeri	2	captive
591	German	female	3,0	819	16,3	163	12,1	8,4	32,9	40,2	pyramiding	no deformation	0,19	0,49	0,94	0,99	0,62	boettgeri	2	captive
592	German	male	3,0	768	15,1	151	12,5	7,8	32,5	39,7	no pyramiding	no deformation	0,22	0,52	1,00	1,02	0,60	boettgeri		captive
593	German	male	3,5	1049	17,1	171	13,4	8,9	36,7	44,3	no pyramiding	no deformation	0,21	0,51	0,98	1,02	0,65	boettgeri		captive
594	German	female	3,5	1112	16,5	165	13,9	9,2	37,2	42,6	pyramiding	no deformation	0,25	0,53	1,01	1,03	0,70	boettgeri	2	captive
595	German	female	3,5	1057	16,3	163	14,1	8,7	37,2	42,2	pyramiding	no deformation	0,24	0,53	1,01	1,03	0,67	boettgeri	2	captive
596	German	female	3,0	966	16,6	166	13,6	8,2	35,4	41,9	pyramiding	no deformation	0,21	0,52	1,00	1,01	0,65	boettgeri	3	captive
597	German	male	2,5	876	17,7	177	13,8	6,9	33,9	43,5	no pyramiding	deformation	0,16	0,52	1,00	0,98	0,59	boettgeri	3	captive
598	German	male	2,5	653	16,3	163	12,8	6,2	31,5	39,3	no pyramiding	deformation	0,15	0,50	0,96	0,97	0,53	boettgeri	3	captive
599	German	female	3,0	760	15,7	157	12,7	7,8	33,0	39,4	pyramiding	no deformation	0,20	0,49	0,93	0,99	0,58	boettgeri	2	captive
600	German	female	3,0	976	16,6	166	14,3	8,5	37,0	42,0	pyramiding	no deformation	0,21	0,48	0,92	1,01	0,63	boettgeri	2	captive
601	German	female	2,5	914	16,2	162	13,7	8,3	35,4	41,6	pyramiding	no deformation	0,21	0,50	0,95	1,01	0,62	boettgeri	2	captive
602	German	female	3,0	1222	17,8	178	15,0	8,9	38,9	45,4	pyramiding	no deformation	0,22	0,51	0,98	1,01	0,69	boettgeri	2	captive
603	German	female	3,0	1171	17,0	170	14,1	9,2	38,4	44,7	pyramiding	no deformation	0,24	0,53	1,01	1,03	0,68	boettgeri	2	captive

636	German	male	3,0	590	13,8	138	11,6	7,8	31,2	35,1	pyramiding	deformation	0,22	0,48	0,91	1,02	0,54	boettgeri	3	captive
637	German	male	3,5	1057	16,8	167	14,1	8,9	37,8	44,0	pyramiding	no deformation	0,22	0,50	0,96	1,03	0,64	boettgeri	2	captive
638	German	male	3,5	1001	17,0	170	14,0	8,3	36,6	43,8	no pyramiding	no deformation	0,20	0,51	0,97	1,01	0,62	boettgeri	3	captive
639	German	female	3,5	3163	26,7	267	19,1	11,7	52,2	65,0	no pyramiding	no deformation	0,17	0,53	1,01	0,99	0,93	boettgeri	5	captive
640	German	female	4,0	3387	24,8	248	20,9	11,4	53,9	63,0	no pyramiding	deformation	0,22	0,57	1,09	1,03	1,00	boettgeri	5	captive
641	German	female	3,0	1060	17,4	174	13,5	8,6	36,8	44,3	no pyramiding	no deformation	0,20	0,52	1,00	1,00	0,65	hermanni	5	captive
642	German	female	3,0	1037	15,9	159	13,1	8,7	36,0	43,6	pyramiding	deformation	0,26	0,58	1,10	1,04	0,66	boettgeri	4	captive
643	German	female	3,0	1289	18,4	184	14,2	9,2	38,5	45,8	no pyramiding	no deformation	0,21	0,54	1,02	1,01	0,73	hermanni	6	captive
644	German	female	3,0	1632	19,9	199	16,4	10,0	43,2	51,0	no pyramiding	no deformation	0,21	0,50	0,96	1,01	0,74	boettgeri	4	captive
645	German	female	3,0	2123	22,7	227	16,8	11,4	47,0	56,7	no pyramiding	no deformation	0,18	0,49	0,93	1,00	0,80	boettgeri	5	captive
646	German	female	2,5	2294	23,6	236	16,6	11,0	45,7	57,7	no pyramiding	no deformation	0,17	0,53	1,02	0,99	0,87	boettgeri	5	captive
647	German	female	3,0	1834	22,0	220	15,3	10,0	42,5	53,2	no pyramiding	no deformation	0,17	0,54	1,04	0,99	0,81	boettgeri	6	captive
648	German	female	3,0	2160	22,3	223	16,5	10,8	44,5	55,5	no pyramiding	no deformation	0,19	0,54	1,04	1,01	0,87	boettgeri	5	captive
649	German	female	3,5	1908	21,0	210	16,4	10,8	44,4	55,0	pyramiding	no deformation	0,21	0,51	0,98	1,01	0,78	boettgeri	3	captive
650	German	female	3,5	991	17,5	175	13,1	8,6	35,5	43,8	no pyramiding	no deformation	0,18	0,50	0,96	0,99	0,64	hermanni	3	captive
651	German	female	3,0	924	17,4	174	13,0	8,5	35,3	43,3	no pyramiding	no deformation	0,18	0,48	0,92	0,98	0,60	boettgeri	3	captive
652	German	male	3,0	611	15,0	150	12,4	6,9	31,5	38,2	pyramiding	deformation	0,18	0,48	0,92	0,99	0,51	boettgeri	1	captive
653	German	male	2,5	446	13,0	130	11,1	6,5	29,2	34,2	pyramiding	deformation	0,20	0,48	0,91	1,00	0,45	boettgeri	1	captive
654	German	female	3,0	678	15,2	152	11,8	7,0	31,5	38,6	pyramiding	no deformation	0,19	0,54	1,03	0,99	0,56	boettgeri	1	captive
655	German	male	3,0	357	12,5	125	10,1	5,5	26,0	31,3	pyramiding	deformation	0,18	0,51	0,98	0,98	0,44	boettgeri	1	captive
656	German	female	3,0	503	13,7	137	11,3	6,1	29,1	35,7	pyramiding	no deformation	0,20	0,53	1,02	0,99	0,48	boettgeri	1	captive
657	German	female	2,5	509	14,3	143	11,4	7,0	30,1	36,2	pyramiding	no deformation	0,17	0,45	0,85	0,97	0,47	boettgeri	1	captive
658	German	male	3,0	625	15,4	154	12,6	7,3	32,9	39,8	no pyramiding	no deformation	0,17	0,44	0,84	0,98	0,48	boettgeri	3	captive
659	German	male	3,0	750	15,3	153	12,7	8,1	34,1	39,1	pyramiding	no deformation	0,21	0,48	0,91	1,01	0,56	boettgeri	3	captive
660	German	male	3,0	590	14,2	142	12,1	7,2	31,0	37,2	no pyramiding	no deformation	0,21	0,48	0,91	1,01	0,51	boettgeri	4	captive
661	German	female	3,5	1244	17,4	174	14,1	9,5	38,2	45,4	pyramiding	deformation	0,24	0,53	1,02	1,02	0,72	boettgeri	4	captive
662	German	female	3,0	1377	19,7	197	14,4	9,0	39,1	48,5	no pyramiding	no deformation	0,18	0,54	1,03	0,99	0,73	boettgeri	5	captive
663	German	female	3,0	1360	19,1	191	14,5	8,8	39,4	48,6	pyramiding	no deformation	0,20	0,56	1,07	1,00	0,71	boettgeri	5	captive
664	German	female	3,5	1484	18,4	184	15,6	9,5	41,0	47,7	pyramiding	no deformation	0,24	0,54	1,04	1,03	0,76	boettgeri	2	captive
665	German	male	2,5	1265	19,0	190	14,7	9,0	39,5	46,9	no pyramiding	no deformation	0,18	0,51	0,97	1,00	0,68	boettgeri	4	captive
666	German	female	2,5	706	15,2	152	12,0	7,2	31,6	39,0	no pyramiding	no deformation	0,20	0,54	1,03	1,00	0,57	hermanni	3	captive
693	German	female	2,5	1700	21,1	211	15,3	10,0	41,4	51,6	no pyramiding	no deformation	0,18	0,53	1,01	0,99	0,80	boettgeri	5	captive
694	German	male	3,0	915	16,7	167	13,3	8,2	35,0	41,3	no pyramiding	no deformation	0,20	0,50	0,96	1,01	0,63	boettgeri	4	captive
695	German	female	3,0	1300	19,0	190	14,3	9,2	38,2	47,0	no pyramiding	no deformation	0,19	0,52	0,99	1,00	0,72	boettgeri		captive
696	German	female	3,0	1064	17,6	176	12,8	8,8	34,6	44,0	no pyramiding	no deformation	0,20	0,54	1,03	1,00	0,70	boettgeri		captive
697	German	male	3,5	1097	17,7	177	15,0	7,8	37,7	44,8	no pyramiding	deformation	0,20	0,53	1,01	1,01	0,65	boettgeri	4	captive
698	German	male	3,0	634	14,6	146	12,0	7,4	31,1	37,4	pyramiding	no deformation	0,20	0,49	0,93	1,01	0,55	boettgeri	2	captive
699	German	female	3,0	1842	21,2	212	15,2	10,4	41,8	52,8	no pyramiding	no deformation	0,19	0,55	1,05	1,00	0,83	boettgeri	5	captive
700	German	male	3,0	633	14,6	146	12,0	7,3	31,3	37,2	pyramiding	no deformation	0,20	0,49	0,95	1,01	0,54	boettgeri	2	captive
701	German	female	3,5	1675	20,9	209	15,7	10,2	41,9	51,5	no pyramiding	no deformation	0,18	0,50	0,96	1,00	0,78	boettgeri	5	captive
702	German	male	3,0	461	12,2	122	10,5	6,9	28,2	32,8	no pyramiding	deformation	0,25	0,52	1,00	1,03	0,50	boettgeri	3	captive
703	German	male	3,0	596	14,8	148	11,4	7,2	30,4	37,3	no pyramiding	no deformation	0,18	0,49	0,94	0,99	0,53	boettgeri	1	captive
704	German	male	3,5	1216	18,4	184	14,8	9,0	38,5	47,2	pyramiding	no deformation	0,20	0,50	0,95	1,01	0,67	boettgeri	5	captive
705	German	female	3,0	2243	23,2	232	17,2	11,2	46,3	56,6	no pyramiding	no deformation	0,18	0,50	0,96	1,00	0,86	boettgeri	6	captive
706	German	male	3,0	1439	19,9	199	15,2	9,7	40,8	49,1	no pyramiding	no deformation	0,18	0,49	0,94	1,00	0,72	boettgeri	6	captive
707	German	female	3,5	1610	21,0	210	15,3	10,1	41,8	52,2	no pyramiding	no deformation	0,17	0,50	0,95	0,99	0,74	boettgeri	6	captive
708	German	female	3,5	2235	22,1	221	16,8	10,7	45,7	55,8	no pyramiding	deformation	0,21	0,56	1,07	1,01	0,88	boettgeri	6	captive
709	German	female	3,5	1881	21,1	211	15,7	10,7	42,5	52,9	no pyramiding	no deformation	0,20	0,53	1,01	1,01	0,84	boettgeri	6	captive
710	German	female	3,5	1003	17,2	172	13,1	8,2	34,8	42,5	no pyramiding	no deformation	0,20	0,54	1,04	1,00	0,68	boettgeri	6	captive

711	German	female	3,0	1106	18,0	180	13,3	8,9	37,6	44,6	no pyramiding	no deformation	0,19	0,52	0,99	0,99	0,66	boettgeri	6	captive
712	German	female	3,5	1075	17,6	176	13,3	8,6	35,8	34,4	no pyramiding	no deformation	0,20	0,54	1,02	1,00	0,87	boettgeri	6	captive
713	German	male	3,0	1369	19,5	195	14,5	9,6	39,5	48,8	no pyramiding	no deformation	0,18	0,50	0,96	1,00	0,71	boettgeri	6	captive
714	German	female	3,0	2278	23,4	234	18,2	11,1	47,0	57,5	pyramiding	no deformation	0,18	0,48	0,92	1,00	0,84	boettgeri	6	captive
717	German	female	3,0	386	12,6	126	9,8	6,6	26,6	32,3	pyramiding	no deformation	0,19	0,47	0,90	0,98	0,45	boettgeri	1	captive
718	German	female	2,5	888	17,7	177	13,5	8,3	35,7	44,0	no pyramiding	no deformation	0,16	0,45	0,86	0,97	0,57	hermanni	3	captive
719	German	female	2,5	690	15,8	158	12,5	8,2	33,2	40,3	pyramiding	no deformation	0,17	0,43	0,81	0,98	0,52	boettgeri	2	captive
720	German	female	3,0	937	17,7	177	13,3	8,8	35,5	44,5	pyramiding	no deformation	0,17	0,45	0,86	0,98	0,59	boettgeri	2	captive
721	German	female	3,0	667	15,9	159	12,2	7,8	32,2	39,8	pyramiding	no deformation	0,17	0,44	0,84	0,97	0,52	hermanni	2	captive
722	German	male	3,0	830	16,2	162	12,2	7,8	32,7	41,2	no pyramiding	no deformation	0,20	0,54	1,03	1,00	0,62	boettgeri	6	captive
723	German	male	2,5	576	15,0	150	11,5	7,3	31,0	37,0	no pyramiding	no deformation	0,17	0,46	0,87	0,98	0,50	hermanni	4	captive
724	German	female	3,5	2025	21,6	216	16,3	10,8	44,3	54,0	pyramiding	no deformation	0,20	0,53	1,02	1,01	0,85	boettgeri	3	captive
725	German	female	3,0	1760	20,2	202	15,5	11,1	42,4	51,1	pyramiding	no deformation	0,21	0,51	0,97	1,02	0,81	boettgeri	3	captive
726	German	female	2,5	1159	18,4	184	13,7	9,1	37,2	45,5	pyramiding	no deformation	0,19	0,51	0,96	0,99	0,68	boettgeri	3	captive
727	German	female	3,5	1571	19,4	194	14,3	9,9	40,0	48,7	pyramiding	no deformation	0,22	0,57	1,09	1,02	0,81	boettgeri	3	captive
728	German	female	3,5	1710	19,9	199	15,4	10,9	42,7	51,0	pyramiding	no deformation	0,22	0,51	0,98	1,02	0,79	boettgeri	5	captive
729	German	female	3,0	1095	16,6	166	14,0	8,3	37,2	42,7	no pyramiding	no deformation	0,24	0,57	1,08	1,03	0,69	boettgeri	4	captive
730	German	female	3,0	1268	18,7	187	14,5	9,3	38,2	47,4	pyramiding	no deformation	0,19	0,50	0,96	1,00	0,70	hermanni	4	captive
731	German	female	3,0	839	16,3	163	12,1	8,0	32,6	40,6	no pyramiding	no deformation	0,19	0,53	1,02	0,99	0,63	hermanni	4	captive
732	German	female	3,0	870	17,0	170	13,0	7,7	34,1	42,9	no pyramiding	no deformation	0,18	0,51	0,98	0,98	0,59	boettgeri	5	captive
733	German	female	3,0	1569	20,0	200	15,4	10,5	41,9	50,0	pyramiding	no deformation	0,20	0,49	0,93	1,00	0,75	hermanni	4	captive
734	German	female	3,5	1330	19,1	191	14,1	9,4	38,5	47,2	pyramiding	no deformation	0,19	0,53	1,00	1,00	0,73	boettgeri	6	captive
735	German	female	3,0	2270	23,1	231	16,9	11,2	45,5	57,2	no pyramiding	no deformation	0,18	0,52	0,99	1,00	0,87	boettgeri	5	captive
736	German	female	3,5	2070	22,2	222	16,1	10,9	44,7	55,2	pyramiding	no deformation	0,19	0,53	1,01	1,00	0,84	boettgeri	5	captive
737	German	female	3,0	2002	21,5	215	16,1	10,5	44,0	54,5	pyramiding	no deformation	0,20	0,55	1,05	1,01	0,83	boettgeri	4	captive
738	German	female	3,0	1150	18,7	187	13,7	8,9	37,1	45,9	pyramiding	no deformation	0,18	0,50	0,96	0,99	0,68	hermanni	5	captive
739	German	male	3,0	636	14,5	145	11,4	7,7	31,9	36,9	no pyramiding	no deformation	0,21	0,50	0,95	1,01	0,54	hermanni	5	captive
740	German	female	3,0	1900	21,3	213	16,1	10,3	42,8	53,2	pyramiding	no deformation	0,20	0,54	1,03	1,01	0,83	boettgeri	3	captive
741	German	male	3,0	1126	17,9	179	13,7	9,0	37,7	44,8	no pyramiding	no deformation	0,20	0,51	0,97	1,01	0,67	hermanni	6	captive
742	German	male	2,5	896	17,3	173	12,5	8,3	34,3	42,8	no pyramiding	no deformation	0,17	0,50	0,95	0,99	0,61	hermanni	6	captive
743	German	male	2,5	570	15,6	156	11,4	6,7	29,4	38,0	no pyramiding	no deformation	0,15	0,48	0,91	0,96	0,51	boettgeri		captive
744	German	male	3,0	1136	17,6	176	14,0	9,5	39,1	45,5	no pyramiding	no deformation	0,21	0,49	0,93	1,02	0,64	hermanni		captive
745	German	male	3,5	644	15,2	152	11,0	7,0	29,5	38,1	no pyramiding	no deformation	0,18	0,55	1,05	0,99	0,57	boettgeri	6	captive
746	German	male	3,5	1560	19,7	197	15,8	9,3	41,6	49,8	pyramiding	no deformation	0,20	0,54	1,03	1,02	0,75	boettgeri	6	captive
747	German	male	3,5	814	15,7	157	12,0	7,7	32,6	39,2	no pyramiding	no deformation	0,21	0,56	1,07	1,01	0,64	hermanni	6	captive
748	German	male	3,5	820	15,9	159	12,5	7,5	33,2	39,8	no pyramiding	no deformation	0,20	0,55	1,05	1,01	0,62	hermanni	3	captive
749	German	male	3,0	740	15,4	154	12,6	7,7	32,3	38,3	no pyramiding	no deformation	0,20	0,50	0,95	1,01	0,60	boettgeri	3	captive
757	German	female	2,5	872	17,1	171	12,6	8,4	34,0	41,7	pyramiding	no deformation	0,17	0,48	0,92	0,98	0,62	boettgeri	3	captive
758	German	female	2,5	1014	18,0	180	12,7	8,8	34,3	43,5	pyramiding	no deformation	0,17	0,50	0,96	0,98	0,68	boettgeri	3	captive
759	German	female	3,0	981	17,3	173	12,9	8,3	34,6	42,4	pyramiding	no deformation	0,19	0,53	1,01	0,99	0,67	boettgeri	3	captive
760	German	female	3,5	994	17,3	173	12,7	8,5	34,8	42,4	no pyramiding	no deformation	0,19	0,53	1,02	0,99	0,67	boettgeri	3	captive
761	German	female	2,5	770	16,1	161	12,5	8,1	32,9	39,7	pyramiding	no deformation	0,18	0,47	0,90	0,99	0,59	boettgeri	2	captive
762	German	female	3,5	936	17,1	171	13,4	8,4	35,3	42,0	pyramiding	no deformation	0,19	0,49	0,93	0,99	0,63	boettgeri	3	captive
763	German	female	3,0	888	17,0	170	13,0	8,0	34,2	41,4	no pyramiding	no deformation	0,18	0,50	0,96	0,99	0,63	boettgeri	2	captive
764	German	female	3,0	682	15,8	158	11,6	7,3	30,7	38,2	no pyramiding	no deformation	0,17	0,51	0,98	0,98	0,58	boettgeri	1	captive
765	German	female	3,0	631	15,2	152	11,4	7,2	30,1	37,1	pyramiding	no deformation	0,18	0,51	0,97	0,98	0,57	boettgeri	1	captive
766	German	female	3,5	817	16,4	164	12,0	8,0	32,7	40,4	pyramiding	no deformation	0,19	0,52	0,99	0,99	0,62	boettgeri	3	captive
767	German	female	2,5	905	17,5	175	12,9	8,1	34,2	42,2	pyramiding	no deformation	0,17	0,49	0,95	0,98	0,63	boettgeri	3	captive
784	German	male	3,0	304	12,9	129	9,7	5,7	25,1	31,4	no pyramiding	no deformation	0,14	0,43	0,81	0,93	0,39	boettgeri	0	captive

785	German	female	3,5	280	12,0	120	8,9	5,7	24,1	29,5	pyramiding	no deformation	0,16	0,46	0,88	0,95	0,39	boettgeri	1	captive
786	German	male	3,5	280	12,0	120	8,9	5,7	24,1	29,5	pyramiding	no deformation	0,16	0,46	0,88	0,95	0,39	boettgeri	0	captive
787	German	female	3,0	192	10,6	106	7,9	5,0	21,3	25,9	pyramiding	no deformation	0,16	0,46	0,88	0,94	0,35	hermanni	1	captive
788	German	female	3,5	1306	18,3	183	13,3	9,4	37,3	45,9	no pyramiding	no deformation	0,21	0,57	1,09	1,01	0,76	boettgeri	3	captive
789	German	male	3,0	753	16,4	164	12,9	8,0	33,7	40,4	no pyramiding	no deformation	0,17	0,44	0,85	0,98	0,55	boettgeri	3	captive
790	German	female	3,5	1149	18,7	187	13,9	8,8	37,3	45,6	no pyramiding	no deformation	0,18	0,50	0,96	0,99	0,68	boettgeri	3	captive
791	German	female	3,5	1481	20,4	204	14,3	9,4	39,3	49,9	no pyramiding	no deformation	0,17	0,54	1,03	0,99	0,76	boettgeri	3	captive
792	German	female	3,0	961	17,3	173	18,9	9,0	35,3	43,4	no pyramiding	no deformation	0,19	0,33	0,62	0,99	0,63	hermanni	2	captive
793	German	female	3,0	281	11,1	111	8,6	5,6	23,3	28,2	no pyramiding	no deformation	0,21	0,53	1,00	0,99	0,43	boettgeri	0	captive
794	German	female	3,0	416	13,0	130	9,5	6,8	26,8	32,8	no pyramiding	no deformation	0,19	0,50	0,95	0,98	0,47	boettgeri	0	captive
797	German	male	3,0	750	15,6	156	12,5	7,9	32,8	39,4	no pyramiding	no deformation	0,20	0,49	0,93	1,00	0,58	hermanni	3	captive
798	German	female	3,0	1534	19,7	197	15,8	9,0	41,4	49,5	no pyramiding	no deformation	0,20	0,55	1,05	1,01	0,75	boettgeri	3	captive
799	German	female	2,5	1180	18,8	188	13,6	8,9	36,5	45,5	pyramiding	no deformation	0,18	0,52	0,99	0,99	0,71	boettgeri	3	captive
800	German	male	2,5	1102	17,6	176	14,3	8,7	37,7	44,4	pyramiding	no deformation	0,20	0,50	0,96	1,01	0,66	boettgeri	4	captive
801	German	female	2,5	1814	21,1	211	15,3	10,0	42,1	53,2	pyramiding	no deformation	0,19	0,56	1,07	1,00	0,81	boettgeri	3	captive
802	German	female	3,5	1500	18,6	186	15,1	9,9	40,1	48,2	no pyramiding	no deformation	0,23	0,54	1,03	1,02	0,78	boettgeri	3	captive
806	German	male	2,0	1470	21,1	211	15,7	9,3	41,2	50,9	no pyramiding	no deformation	0,16	0,48	0,91	0,98	0,70	boettgeri	6	captive
807	German	male	2,0	415	12,2	122	9,8	6,4	26,4	31,8	no pyramiding	no deformation	0,23	0,54	1,04	1,02	0,49	hermanni	4	captive
808	German	male	3,0	653	14,6	146	11,8	7,1	30,6	37,2	no pyramiding	no deformation	0,21	0,53	1,02	1,01	0,57	boettgeri	4	captive
809	German	male	2,0	724	16,1	161	12,2	7,3	31,2	39,6	pyramiding	no deformation	0,17	0,50	0,96	0,99	0,59	boettgeri	3	captive
810	German	male	3,0	716	14,5	145	12,8	7,5	31,4	37,2	pyramiding	no deformation	0,23	0,51	0,98	1,03	0,61	boettgeri	2	captive
811	German	male	3,0	855	16,1	161	12,7	7,9	33,4	40,0	no pyramiding	no deformation	0,20	0,53	1,01	1,01	0,64	boettgeri		captive
812	German	male	2,5	692	15,6	156	11,8	7,6	31,1	38,7	pyramiding	no deformation	0,18	0,49	0,94	0,99	0,57	boettgeri	2	captive
813	German	male	3,0	534	14,2	142	10,9	6,8	29,4	35,3	no pyramiding	no deformation	0,19	0,51	0,97	0,99	0,51	boettgeri	5	captive
815	German	male	3,0	789	16,1	161	12,2	7,9	32,6	40,1	pyramiding	no deformation	0,19	0,51	0,97	1,00	0,60	boettgeri	4	captive
817	German	male	3,0	501	13,3	133	10,7	6,9	28,6	33,9	pyramiding	no deformation	0,21	0,51	0,97	1,01	0,52	boettgeri		captive
818	German	male	3,0	417	12,7	127	10,4	6,0	27,0	31,8	no pyramiding	no deformation	0,20	0,53	1,00	1,00	0,49	boettgeri	1	captive
820	German	male	3,5	1005	17,1	171	13,3	8,3	35,5	42,6	no pyramiding	no deformation	0,20	0,53	1,02	1,01	0,66	boettgeri	6	captive
823	German	male	4,0	867	15,2	152	13,4	7,7	34,5	39,8	pyramiding	no deformation	0,25	0,55	1,06	1,04	0,63	boettgeri	5	captive
825	German	male	3,0	882	15,9	159	13,4	8,3	34,7	40,8	pyramiding	no deformation	0,22	0,50	0,95	1,02	0,62	boettgeri	3	captive
827	German	male	3,0	1163	17,4	174	15,5	8,5	38,7	44,5	no pyramiding	deformation	0,22	0,51	0,97	1,02	0,68	boettgeri	4	captive
828	German	male	3,0	738	15,8	158	12,5	7,6	32,6	39,9	pyramiding	no deformation	0,19	0,49	0,94	1,00	0,57	boettgeri	5	captive
829	German	female	2,5	1273	20,0	200	15,2	8,7	39,8	48,8	pyramiding	no deformation	0,16	0,48	0,92	0,97	0,66	boettgeri	2	captive
830	German	female	3,5	1631	20,1	201	15,8	9,1	42,5	49,5	no pyramiding	no deformation	0,20	0,56	1,08	1,01	0,78	boettgeri	6	captive
831	German	male	2,5	495	14,0	140	10,5	6,5	28,6	34,5	no pyramiding	no deformation	0,18	0,52	0,99	0,98	0,50	boettgeri	1	captive
833	German	female	3,5	1274	19,0	190	13,9	8,8	37,5	46,5	no pyramiding	no deformation	0,19	0,55	1,05	0,99	0,73	boettgeri	3	captive
834	German	female	4,0	1706	20,0	200	14,8	10,1	40,9	50,1	no pyramiding	no deformation	0,21	0,57	1,09	1,01	0,83	boettgeri	6	captive
835	German	female	4,0	1392	19,8	198	13,8	9,2	37,8	48,0	no pyramiding	no deformation	0,18	0,55	1,06	0,99	0,77	boettgeri	3	captive
836	German	male	3,0	657	14,7	147	12,1	7,3	31,7	36,8	no pyramiding	no deformation	0,21	0,51	0,97	1,01	0,56	boettgeri	4	captive
850	German	male	3,0	838	16,5	165	12,9	8,5	34,3	41,4	no pyramiding	no deformation	0,19	0,46	0,88	1,00	0,59	boettgeri	4	captive
851	German	male	3,5	737	16,6	166	12,3	8,0	33,0	40,8	no pyramiding	no deformation	0,16	0,45	0,86	0,98	0,55	boettgeri	4	captive
852	German	male	4,0	747	15,9	159	13,0	8,1	34,3	40,2	no pyramiding	no deformation	0,19	0,45	0,85	1,00	0,54	boettgeri	3	captive
853	German	male	3,5	435	13,3	133	10,6	7,0	28,3	33,6	no pyramiding	no deformation	0,18	0,44	0,84	0,99	0,46	boettgeri	3	captive
855	German	female	3,0	1604	20,9	209	14,6	10,3	41,6	51,6	pyramiding	no deformation	0,18	0,51	0,97	0,99	0,75	boettgeri	3	captive
856	German	female	3,0	1297	20,3	203	13,8	9,7	38,0	49,1	pyramiding	no deformation	0,16	0,48	0,91	0,97	0,70	boettgeri	3	captive
857	German	female	3,0	1047	17,5	175	13,4	8,9	37,0	44,2	pyramiding	no deformation	0,20	0,50	0,96	1,00	0,64	boettgeri	4	captive
858	German	female	3,0	1535	20,4	204	14,9	10,0	40,6	50,9	no pyramiding	no deformation	0,18	0,51	0,97	0,99	0,74	boettgeri	6	captive
859	German	female	4,0	1203	18,8	188	13,9	9,1	37,3	46,1	no pyramiding	no deformation	0,18	0,51	0,97	0,99	0,70	boettgeri	6	captive
860	German	female	3,0	1631	21,1	211	14,7	10,4	40,8	51,5	no pyramiding	no deformation	0,17	0,51	0,97	0,99	0,78	boettgeri	6	captive

861	German	female	3,0	1341	19,7	197	13,5	9,3	37,6	47,9	no pyramiding	no deformation	0,18	0,54	1,04	0,99	0,74	boettgeri	5	captive
862	German	female	3,5	1367	19,4	194	13,9	9,6	38,6	47,9	no pyramiding	no deformation	0,19	0,53	1,01	1,00	0,74	boettgeri	5	captive
863	German	female	2,5	909	17,0	170	13,2	8,4	36,0	42,7	pyramiding	no deformation	0,19	0,48	0,92	0,99	0,59	boettgeri	4	captive
864	German	female	3,0	407	13,0	130	10,1	6,2	27,0	32,8	no pyramiding	no deformation	0,19	0,50	0,95	0,97	0,46	boettgeri	1	captive
865	German	female	3,0	411	13,2	132	9,8	6,3	26,3	32,8	no pyramiding	no deformation	0,18	0,50	0,96	0,97	0,48	boettgeri	1	captive
866	German	female	3,0	334	12,5	125	9,4	5,8	24,9	30,8	no pyramiding	no deformation	0,17	0,49	0,94	0,96	0,44	boettgeri	1	captive
874	German	female	3,0	866	16,1	161	13,6	7,8	34,6	40,7	pyramiding	no deformation	0,21	0,51	0,97	1,00	0,61	boettgeri	2	captive
875	German	male	3,0	726	15,1	151	13,0	7,1	33,0	38,0	pyramiding	no deformation	0,21	0,52	0,99	1,01	0,58	boettgeri	2	captive
876	German	female	3,5	2070	21,2	212	16,6	11,1	44,4	53,0	pyramiding	no deformation	0,22	0,53	1,01	1,02	0,88	boettgeri	3	captive
877	German	female	3,5	3090	24,6	246	18,2	12,7	50,1	62,4	pyramiding	no deformation	0,21	0,54	1,04	1,01	0,99	boettgeri	3	captive
878	German	female	4,5	2660	23,3	233	17,0	11,4	46,7	57,3	no pyramiding	no deformation	0,21	0,59	1,13	1,01	0,99	boettgeri	6	captive
879	German	male	4,0	1200	17,4	174	14,6	8,8	38,8	43,4	no pyramiding	no deformation	0,23	0,54	1,03	1,03	0,71	boettgeri	5	captive
880	German	male	2,5	779	16,6	166	12,9	7,8	33,4	40,6	pyramiding	no deformation	0,17	0,47	0,89	0,98	0,57	boettgeri	2	captive
881	German	male	3,0	774	15,9	158	12,6	7,9	33,2	40,0	pyramiding	no deformation	0,19	0,49	0,94	1,00	0,58	boettgeri	3	captive
882	German	male	3,0	703	15,2	152	12,1	7,5	31,7	37,9	no pyramiding	no deformation	0,20	0,51	0,97	1,00	0,59	boettgeri	6	captive
883	German	male	3,5	785	16,6	166	12,8	8,0	34,0	41,3	pyramiding	no deformation	0,17	0,46	0,88	0,98	0,56	boettgeri	3	captive
886	German	male	2,5	697	15,1	151	12,2	8,1	32,6	38,9	pyramiding	no deformation	0,20	0,47	0,89	1,00	0,55	boettgeri	2	captive
888	German	male	3,5	894	16,4	164	12,6	8,4	34,3	42,4	pyramiding	no deformation	0,20	0,52	0,98	1,01	0,61	boettgeri	2	captive
889	German	male	3,0	700	15,1	151	12,1	7,3	32,0	38,0	no pyramiding	no deformation	0,20	0,52	1,00	1,00	0,58	boettgeri	5	captive
890	German	male	3,0	700	14,5	145	12,0	7,8	32,1	38,5	no pyramiding	no deformation	0,23	0,52	0,99	1,02	0,57	boettgeri	2	captive
891	German	male	3,0	731	15,5	155	12,3	7,5	32,2	39,3	no pyramiding	no deformation	0,20	0,51	0,98	1,00	0,58	boettgeri	4	captive
892	German	male	2,5	567	13,7	137	11,6	6,7	30,5	35,3	no pyramiding	deformation	0,22	0,53	1,02	1,01	0,53	boettgeri	6	captive
893	German	male	2,5	598	15,1	151	11,8	7,5	31,0	37,6	pyramiding	no deformation	0,17	0,45	0,85	0,98	0,51	boettgeri	2	captive
895	German	male	3,0	635	14,9	149	11,7	7,0	30,6	37,0	no pyramiding	no deformation	0,19	0,52	0,99	0,99	0,56	boettgeri	2	captive
897	German	male	3,0	493	13,3	133	10,8	7,0	28,8	34,3	no pyramiding	no deformation	0,21	0,49	0,94	1,00	0,50	hermanni	3	captive
898	German	male	3,0	505	12,7	127	11,1	6,3	29,4	33,2	pyramiding	deformation	0,25	0,57	1,09	1,03	0,52	boettgeri	2	captive
901	German	male	2,0	706	16,3	163	12,6	7,8	33,2	40,9	pyramiding	no deformation	0,16	0,44	0,84	0,97	0,52	hermanni	25	captive
902	German	male	2,5	531	14,6	146	11,0	6,7	28,7	36,5	pyramiding	no deformation	0,17	0,49	0,94	0,97	0,51	boettgeri	1	captive
903	German	male	3,0	579	14,0	140	11,3	7,2	30,0	36,5	pyramiding	deformation	0,21	0,51	0,97	1,01	0,53	boettgeri	1	captive
904	German	male	2,5	465	13,1	131	10,2	6,9	27,9	33,7	no pyramiding	no deformation	0,21	0,50	0,96	1,00	0,49	boettgeri	3	captive
905	German	male	3,0	669	14,9	149	11,9	7,8	31,1	37,5	no pyramiding	no deformation	0,20	0,48	0,92	1,00	0,57	boettgeri	2	captive
907	German	male	3,0	707	15,0	150	11,9	7,9	32,4	38,7	no pyramiding	no deformation	0,21	0,50	0,96	1,01	0,56	hermanni	2	captive
908	German	female	3,5	440	12,5	125	10,8	5,9	27,3	31,7	no pyramiding	no deformation	0,23	0,55	1,06	1,00	0,51	boettgeri	2	captive
909	German	male	3,5	1127	17,9	179	14,1	8,3	37,3	44,6	no pyramiding	no deformation	0,20	0,54	1,03	1,01	0,68	boettgeri	6	captive
910	German	male	4,0	1230	16,9	169	15,5	8,6	40,0	43,5	no pyramiding	no deformation	0,25	0,55	1,04	1,04	0,71	boettgeri	5	captive
911	German	male	4,0	892	15,8	158	13,3	8,3	34,7	40,5	no pyramiding	no deformation	0,23	0,51	0,98	1,02	0,63	boettgeri	2	captive
912	German	male	3,0	666	15,0	150	12,5	7,5	31,7	37,8	pyramiding	no deformation	0,20	0,47	0,90	1,00	0,56	boettgeri	3	captive
913	German	male	3,0	795	16,4	164	12,3	8,0	32,8	40,7	no pyramiding	no deformation	0,18	0,49	0,94	0,99	0,60	boettgeri	3	captive
914	German	male	2,5	1456	20,1	201	15,8	9,1	40,8	50,3	no pyramiding	no deformation	0,18	0,50	0,96	1,00	0,71	boettgeri	5	captive
915	German	male	3,0	681	15,1	151	11,7	7,9	31,9	38,8	no pyramiding	no deformation	0,20	0,49	0,93	1,00	0,55	boettgeri	5	captive
916	German	male	3,0	650	14,3	143	12,2	7,5	31,5	37,2	pyramiding	no deformation	0,22	0,50	0,95	1,01	0,55	boettgeri	2	captive
918	German	male	3,5	1082	18,0	180	14,3	8,5	37,7	45,1	no pyramiding	no deformation	0,19	0,49	0,94	1,00	0,64	hermanni	5	captive
919	German	male	2,0	709	16,1	161	13,5	7,8	34,5	40,5	no pyramiding	no deformation	0,17	0,42	0,80	0,98	0,51	boettgeri	3	captive
920	German	male	2,5	830	17,1	171	12,7	8,3	35,2	41,8	no pyramiding	no deformation	0,17	0,46	0,88	0,98	0,56	boettgeri	4	captive
921	German	male	3,0	676	15,0	150	11,6	7,4	31,2	38,2	no pyramiding	no deformation	0,20	0,53	1,00	1,00	0,57	boettgeri	3	captive
922	German	female	3,0	1437	19,9	199	15,4	8,8	40,4	48,9	pyramiding	no deformation	0,18	0,53	1,02	0,99	0,73	boettgeri		captive
923	German	female	3,0	941	15,5	155	14,1	7,7	35,7	40,0	no pyramiding	deformation	0,25	0,56	1,07	1,03	0,66	boettgeri	5	captive
924	German	female	3,0	900	15,3	153	13,5	8,3	35,8	40,2	no pyramiding	no deformation	0,25	0,52	1,00	1,03	0,63	boettgeri	5	captive
925	German	female	3,5	1149	18,1	181	13,9	8,8	36,3	45,0	pyramiding	no deformation	0,19	0,52	0,99	0,99	0,70	boettgeri	3	captive

926	German	female	3,0	1450	19,5	195	15,1	9,4	39,5	49,3	pyramiding	no deformation	0,20	0,52	1,00	1,00	0,74	boettgeri	3	captive
927	German	female	3,0	1188	18,8	188	14,1	8,3	36,8	46,2	no pyramiding	no deformation	0,18	0,54	1,03	0,98	0,70	boettgeri	3	captive
928	German	female	3,0	803	16,2	162	12,7	7,6	32,5	40,7	pyramiding	no deformation	0,19	0,51	0,98	0,99	0,61	boettgeri	3	captive
929	German	female	4,0	1183	17,9	179	14,3	8,2	36,4	45,0	pyramiding	no deformation	0,21	0,56	1,08	1,00	0,72	boettgeri	3	captive
930	German	female	3,0	413	12,6	126	10,2	6,6	27,0	32,7	no pyramiding	no deformation	0,21	0,49	0,93	0,99	0,47	boettgeri	1	captive
931	German	female	2,5	358	12,4	124	10,1	6,3	26,4	31,2	pyramiding	no deformation	0,19	0,45	0,87	0,97	0,43	boettgeri	1	captive
932	German	male	3,0	1237	18,3	183	15,5	8,4	40,1	46,8	no pyramiding	no deformation	0,20	0,52	0,99	1,01	0,66	boettgeri	2	captive
933	German	male	3,0	433	12,2	122	10,2	6,4	27,4	31,9	no pyramiding	no deformation	0,24	0,54	1,04	1,02	0,50	boettgeri	1	captive
934	German	male	3,0	821	16,0	160	13,3	7,8	34,4	40,5	no pyramiding	no deformation	0,20	0,49	0,94	1,00	0,59	boettgeri	5	captive
935	German	male	3,5	1002	18,8	188	13,9	8,2	36,2	44,5	no pyramiding	no deformation	0,15	0,47	0,89	0,97	0,62	boettgeri	2	captive
938	German	male	3,0	633	15,2	152	11,8	7,2	31,2	37,1	pyramiding	no deformation	0,18	0,49	0,94	0,98	0,55	boettgeri	1	captive
939	German	male	3,0	673	14,7	147	11,7	7,3	31,4	37,3	no pyramiding	no deformation	0,21	0,54	1,02	1,01	0,57	boettgeri	2	captive
940	German	male	3,0	684	14,7	147	12,6	7,6	32,6	38,3	no pyramiding	no deformation	0,22	0,49	0,93	1,01	0,55	boettgeri	4	captive
1152	French	female	3,0	539	14,3	143	11,2	7,0	28,7	35,5	no pyramiding	no deformation	0,18	0,48	0,92	0,98	0,53	hermanni	2	wild
1153	French	female	3,0	650	15,3	153	11,5	8,3	32,7	38,5	no pyramiding	no deformation	0,18	0,45	0,85	0,98	0,52	hermanni	3	wild
1154	French	male	3,0	576	14,9	149	11,6	6,7	30,5	36,5	no pyramiding	no deformation	0,17	0,50	0,95	0,98	0,52	hermanni	2	wild
1155	French	female	3,0	614	14,6	146	11,0	7,1	30,6	36,0	no pyramiding	no deformation	0,20	0,53	1,02	0,99	0,56	hermanni	2	wild
1156	French		3,0	261	10,1	101	8,3	5,6	22,5	26,2	no pyramiding	no deformation	0,25	0,55	1,05	1,02	0,44	hermanni		wild
1157	French	female	3,0	820	17,1	171	12,5	8,1	34,2	42,0	no pyramiding	no deformation	0,16	0,47	0,90	0,97	0,57	hermanni	3	wild
1158	French	female	3,0	914	17,5	175	13,1	8,6	36,1	43,3	no pyramiding	no deformation	0,17	0,46	0,89	0,98	0,58	hermanni	3	wild
1159	French	female	3,0	1011	17,4	174	12,7	8,3	34,9	42,7	no pyramiding	no deformation	0,19	0,55	1,05	0,99	0,68	hermanni	3	wild
1160	French	female	3,0	769	16,0	160	11,7	7,9	32,1	40,0	pyramiding	no deformation	0,19	0,52	1,00	0,99	0,60	hermanni	3	wild
1161	French	female	3,0	778	15,7	157	11,8	7,6	32,0	38,5	no pyramiding	no deformation	0,20	0,55	1,06	1,00	0,63	hermanni	3	wild
1162	French	male	3,0	439	12,7	127	10,4	6,9	28,0	33,0	no pyramiding	no deformation	0,21	0,48	0,92	1,01	0,48	hermanni	2	wild
1163	French	female	3,0	913	16,8	168	12,3	9,0	33,4	41,5	no pyramiding	no deformation	0,19	0,49	0,93	0,99	0,66	hermanni	3	wild
1165	French	female	3,0	857	16,1	161	12,5	7,9	34,0	40,2	no pyramiding	no deformation	0,21	0,54	1,03	1,00	0,63	hermanni	3	wild
1166	French	female	3,0	459	12,5	125	9,8	7,1	27,0	32,6	no pyramiding	no deformation	0,24	0,53	1,02	1,01	0,52	hermanni	2	wild
1167	French	female	4,0	951	17,3	173	12,8	8,6	35,5	42,6	no pyramiding	no deformation	0,19	0,50	0,96	0,99	0,63	hermanni	3	wild
1168	French		3,0	397	12,1	121	9,4	6,5	25,9	30,7	no pyramiding	no deformation	0,23	0,54	1,03	1,00	0,50	hermanni		wild
1169	French	female	3,0	737	14,9	149	11,1	7,9	31,0	37,7	no pyramiding	no deformation	0,22	0,57	1,08	1,01	0,63	hermanni	2	wild
1170	French	male	3,0	581	14,8	148	11,0	7,1	29,6	36,6	no pyramiding	no deformation	0,18	0,51	0,97	0,99	0,54	hermanni	2	wild
1171	French	male	3,0	476	13,7	137	10,3	6,8	27,6	33,9	no pyramiding	no deformation	0,19	0,50	0,96	0,99	0,51	hermanni	2	wild
1172	French	male	3,0	365	13,0	130	10,5	6,6	28,0	32,6	no pyramiding	no deformation	0,17	0,40	0,77	0,97	0,40	hermanni	2	wild
1173	French	female	3,0	644	14,8	148	11,1	7,7	30,4	37,2	no pyramiding	no deformation	0,20	0,51	0,97	0,99	0,57	hermanni	2	wild
1174	French	female	3,0	762	15,8	158	10,7	8,2	32,0	39,3	no pyramiding	no deformation	0,20	0,55	1,06	0,99	0,61	hermanni	2	wild
1175	French	male	3,0	471	13,3	133	10,4	7,2	28,6	34,0	no pyramiding	no deformation	0,20	0,48	0,91	1,00	0,48	hermanni	2	wild
1176	French	male	3,0	310	11,4	114	9,5	5,9	25,5	29,8	no pyramiding	no deformation	0,21	0,49	0,93	1,00	0,41	hermanni	2	wild
1177	French	female	3,0	654	15,0	150	11,2	7,9	30,5	37,3	no pyramiding	no deformation	0,19	0,50	0,95	0,99	0,57	hermanni	2	wild
1178	French	female	2,0	752	16,1	161	11,6	7,5	31,7	39,3	no pyramiding	no deformation	0,18	0,53	1,02	0,98	0,60	hermanni	5	wild
1179	French	female	3,0	893	16,4	164	12,2	8,4	33,2	41,4	no pyramiding	no deformation	0,20	0,53	1,02	1,00	0,65	hermanni	3	wild
1180	French	female	3,0	688	15,9	159	11,7	8,2	31,5	39,5	no pyramiding	no deformation	0,17	0,45	0,86	0,97	0,55	hermanni	3	wild
1181	French	female	3,0	729	15,7	157	11,6	7,9	31,5	39,1	no pyramiding	no deformation	0,19	0,51	0,97	0,99	0,59	hermanni	2	wild
1182	French	female	3,0	936	16,7	167	13,0	8,3	34,4	41,4	no pyramiding	no deformation	0,20	0,52	1,00	1,00	0,66	hermanni	2	wild
1183	French	female	3,0	763	15,9	159	11,5	7,9	31,4	39,1	no pyramiding	no deformation	0,19	0,53	1,01	0,99	0,62	hermanni	3	wild
1184	French	male	3,0	468	14,2	142	10,4	6,6	28,0	34,5	no pyramiding	no deformation	0,16	0,48	0,92	0,97	0,48	hermanni	2	wild
1185	French	female	3,0	654	15,5	155	11,4	8,1	31,0	38,1	no pyramiding	no deformation	0,17	0,46	0,87	0,98	0,55	hermanni	3	wild
1186	French	female	3,0	843	16,0	160	12,1	8,6	32,5	39,8	no pyramiding	no deformation	0,21	0,51	0,97	1,00	0,65	hermanni	3	wild
1187	French	female	3,0	838	16,9	169	12,1	8,8	32,8	40,8	no pyramiding	no deformation	0,17	0,47	0,90	0,98	0,63	hermanni	2	wild
1188	French	male	3,0	323	12,0	120	9,5	6,5	25,9	30,7	no pyramiding	no deformation	0,19	0,44	0,83	0,99	0,41	hermanni	2	wild

1189	French	female	3,0	729	15,4	154	11,7	7,4	32,0	38,5	no pyramiding	no deformation	0,20	0,55	1,04	1,00	0,59	hermanni	2	wild
1190	French	male	3,0	442	13,4	134	10,6	6,9	28,3	33,5	no pyramiding	no deformation	0,18	0,45	0,86	0,99	0,47	hermanni	2	wild
1191	French	female	3,0	721	15,1	151	10,9	7,8	30,6	38,0	no pyramiding	no deformation	0,21	0,56	1,07	1,00	0,62	hermanni	3	wild
1192	French	male	3,0	588	14,6	146	11,0	7,0	29,0	35,8	no pyramiding	no deformation	0,19	0,52	1,00	1,00	0,57	hermanni	3	wild
1193	French	female	3,0	628	15,1	151	11,2	8,0			no pyramiding	no deformation	0,18	0,47	0,89	0,98		hermanni	3	wild
1194	French	female	3,0	790	16,1	161	11,7	8,4			no pyramiding	no deformation	0,19	0,50	0,96	0,99		hermanni	3	wild
1195	French	female	3,0	858	17,0	170	12,4	8,1	33,5	41,6	no pyramiding	no deformation	0,17	0,50	0,95	0,98	0,62	hermanni	3	wild
1196	French	male	3,0	466	13,5	135	10,3	6,4	27,6	33,4	no pyramiding	no deformation	0,19	0,53	1,01	0,99	0,51	hermanni	2	wild
1197	French	female	3,0	831	15,4	154	11,9	8,3	32,4	38,8	no pyramiding	no deformation	0,23	0,55	1,04	1,02	0,66	hermanni	3	wild
1198	French	male	3,0	527	14,2	142	10,3	6,9	28,0	35,0	no pyramiding	no deformation	0,19	0,52	1,00	0,99	0,54	hermanni	3	wild
1199	French	male	3,0	624	14,4	144	11,3	7,8	29,8	36,6	no pyramiding	no deformation	0,21	0,49	0,94	1,01	0,57	hermanni	3	wild
1200	French	male	3,0	451	13,0	130	9,9	6,8	26,8	32,2	no pyramiding	no deformation	0,21	0,52	0,99	1,01	0,52	hermanni	2	wild
1201	French	male	2,0	434	12,3	123	9,8	6,5	26,5	31,5	no pyramiding	no deformation	0,23	0,55	1,05	1,03	0,52	hermanni	2	wild
1202	French	female	3,0	611	14,7	147	10,9	7,4	29,8	36,9	no pyramiding	no deformation	0,19	0,52	0,99	0,99	0,56	hermanni	2	wild
1203	French	female	3,0	753	15,7	157	11,8	8,3	21,4	38,8	no pyramiding	no deformation	0,19	0,49	0,93	0,99	0,91	hermanni	4	wild
1204	French	male	3,0	331	12,3	123	9,6	6,5	26,0	30,9	no pyramiding	no deformation	0,18	0,43	0,82	0,98	0,41	hermanni	4	wild
1205	French	male	3,0	518	14,4	144	11,2	7,4	29,6	36,0	no pyramiding	no deformation	0,17	0,43	0,83	0,98	0,49	hermanni	4	wild
1206	French	male	3,0	410	12,9	129	10,0	6,5	26,7	32,3	no pyramiding	no deformation	0,19	0,49	0,93	0,99	0,48	hermanni	3	wild
1207	French	male	3,0	392	12,5	125	9,3	6,5	25,5	31,7	no pyramiding	no deformation	0,20	0,52	1,00	1,00	0,48	hermanni	4	wild
1208	French	female	3,0	641	15,2	152	10,9	7,2	37,2	29,6	no pyramiding	no deformation	0,18	0,54	1,02	0,99	0,58	hermanni	1	wild
1209	French	female	3,0	852	16,7	167	12,0	7,3	32,3	40,4	no pyramiding	no deformation	0,18	0,59	1,12	1,00	0,65	hermanni	4	wild
1210	French	female	3,0	713	16,0	160	11,9	7,7	33,0	39,4	no pyramiding	no deformation	0,17	0,49	0,94	0,99	0,55	hermanni	4	wild
1211	French	male	3,0	491	13,1	131	11,5	6,8	28,3	33,0	no pyramiding	no deformation	0,22	0,48	0,92	1,02	0,53	hermanni	3	wild
1212	French	female	4,0	1055	17,4	174	12,9	8,2	34,8	42,6	no pyramiding	no deformation	0,20	0,57	1,09	1,00	0,71	hermanni	5	captive
1213	French	female	3,0	896	15,8	158	11,7	8,4	32,7	39,7	no pyramiding	no deformation	0,23	0,58	1,10	1,02	0,69	hermanni	4	captive
1214	French	male	3,0	455	13,2	132	10,1	6,4	27,4	33,1	no pyramiding	no deformation	0,20	0,53	1,01	0,99	0,50	hermanni	4	captive
1215	French	female	3,0	800	15,0	150	11,6	7,8	31,4	38,7	no pyramiding	no deformation	0,24	0,59	1,12	1,02	0,66	hermanni	4	captive
1216	French	male	2,0	372	13,0	130	10,3	6,5	27,1	31,4	no pyramiding	no deformation	0,17	0,43	0,81	0,96	0,44	hermanni	3	captive
1217	French	male	3,0	305	11,5	115	8,8	5,8	23,6	28,8	no pyramiding	no deformation	0,20	0,52	1,00	0,98	0,45	hermanni	2	captive
1218	French	female	4,0	1027	16,3	163	12,8	8,9	35,0	41,9	no pyramiding	no deformation	0,24	0,55	1,05	1,02	0,70	hermanni	5	captive
1219	French	female	3,0	851	16,2	162	11,7	8,4	32,1	40,7	no pyramiding	no deformation	0,20	0,54	1,03	1,00	0,65	hermanni	4	captive
1220	French	male	3,0	543	13,6	136	10,9	7,4	29,4	35,0	no pyramiding	no deformation	0,22	0,50	0,95	1,00	0,53	hermanni	4	captive
1221	French	female	3,0	795	15,5	155	11,6	8,2	31,6	39,0	no pyramiding	no deformation	0,21	0,54	1,04	1,01	0,65	hermanni	3	captive
1222	French	female	4,0	1283	18,9	189	13,8	10,0	38,2	47,0	no pyramiding	no deformation	0,19	0,49	0,94	1,00	0,71	hermanni	5	captive
1223	French	female	4,0	1335	18,3	183	13,4	8,8	36,7	45,7	no pyramiding	no deformation	0,22	0,61	1,17	1,01	0,80	hermanni	5	captive
1224	French	female	4,0	1072	16,6	166	12,9	9,2	35,4	42,0	no pyramiding	no deformation	0,23	0,54	1,04	1,02	0,72	hermanni	4	captive
1225	French	female	4,0	1139	17,2	172	12,9	8,5	34,6	43,1	no pyramiding	no deformation	0,23	0,60	1,15	1,02	0,76	hermanni	4	captive
1226	French	female	3,0	897	15,9	159	12,2	8,1	32,8	40,0	no pyramiding	no deformation	0,22	0,57	1,09	1,01	0,68	hermanni	4	captive
1227	French	female	4,0	956	16,4	164	12,2	8,1	33,4	40,7	no pyramiding	no deformation	0,22	0,59	1,12	1,01	0,70	hermanni	4	captive
1228	French	female	4,0	967	16,9	169	12,0	8,0	33,1	41,9	no pyramiding	no deformation	0,20	0,60	1,14	1,00	0,70	hermanni	4	captive
1229	French	female	4,0	1019	17,3	173	12,1	8,1	33,5	42,1	no pyramiding	no deformation	0,20	0,60	1,15	1,00	0,72	hermanni	4	captive
1230	French	female	3,0	739	15,2	152	11,7	8,3	32,1	39,1	no pyramiding	no deformation	0,21	0,51	0,97	1,00	0,59	hermanni	4	captive
1231	French	female	3,0	682	15,2	152	11,8	7,3	29,9	37,4	no pyramiding	no deformation	0,20	0,53	1,00	0,99	0,61	hermanni	4	captive
1232	French	female	4,0	1082	17,0	170	12,8	8,4	35,2	42,4	no pyramiding	no deformation	0,22	0,60	1,14	1,01	0,72	hermanni	4	captive
1233	French	female	4,0	954	16,9	169	12,0	8,0	33,0	41,4	no pyramiding	no deformation	0,20	0,59	1,13	1,00	0,70	hermanni	4	captive
1234	French	female	3,0	802	16,5	165	12,5	8,2	33,4	40,7	no pyramiding	no deformation	0,18	0,48	0,91	0,98	0,59	hermanni	4	wild
1235	French	male	2,0	486	13,5	135	10,4	7,0	28,5	34,0	no pyramiding	no deformation	0,20	0,50	0,95	0,99	0,50	hermanni	4	wild
1236	French	male	3,0	248	10,4	104	8,3	5,8	22,5	26,5	no pyramiding	no deformation	0,22	0,50	0,95	1,01		hermanni	1	wild
1239	French	female	3,0	391	12,2	122	9,5	6,4	26,0	31,0	no pyramiding	no deformation	0,22	0,53	1,01	1,00	0,49	hermanni	1	wild

1240	French	male	3,0	492	14,0	140	10,3	6,6	27,2	34,4	pyramiding	no deformation	0,18	0,52	0,99	0,99	0,53	hermanni	1	wild
1242	French	male	3,0	305	11,2	112	8,8	6,0	24,1	28,6	no pyramiding	no deformation	0,22	0,52	0,99	1,01	0,44	hermanni	1	wild
1243	French	female	3,0	290	10,7	107	8,8	5,9	27,0	23,5	no pyramiding	no deformation	0,24	0,52	1,00	1,01	0,46	hermanni	1	wild
1247	French	male	3,0	276	10,9	109	8,8	5,9	23,9	27,7	no pyramiding	no deformation	0,21	0,49	0,93	1,01	0,42	hermanni	1	wild
1248	French	male	3,0	232	10,1	101	8,1	5,5	21,7	25,7	no pyramiding	no deformation	0,23	0,52	0,98	1,01	0,42	hermanni	1	wild
1261	French	male	3,0	202	10,0	100	7,5	5,1	20,5	25,0	pyramiding	no deformation	0,20	0,53	1,01	0,99	0,39	hermanni	1	captive
1268	French	male	3,0	207	10,1	101	7,8	5,3	21,0	25,5	no pyramiding	no deformation	0,20	0,50	0,95	0,99	0,39	hermanni	1	captive
1281	German		3,0	346	10,9	109	8,3	6,0	23,5	28,4	pyramiding	deformation	0,27	0,64	1,22	1,03	0,52	boettgeri	1	captive
1284	German		3,0	701	17,7	177	11,5	8,8	32,2	41,5	no pyramiding	no deformation	0,13	0,39	0,75	0,94	0,52	boettgeri	3	captive
1285	German		2,0	967	16,8	168	12,6	7,3	34,1	41,5	pyramiding	deformation	0,20	0,63	1,20	1,00	0,68	boettgeri	1	captive
1286	German		4,0	1482	18,2	182	14,1	9,4	37,3	45,5	no pyramiding	no deformation	0,25	0,61	1,17	1,03	0,87	boettgeri	2	captive
1287	German		2,5	958	17,3	173	12,9	8,3	36,0	42,6	no pyramiding	no deformation	0,19	0,52	0,99	0,99	0,62	boettgeri	1	captive
1289	German		2,5	1248	17,3	173	14,3	9,5	38,3	44,2	no pyramiding	no deformation	0,24	0,53	1,01	1,03	0,74	boettgeri	2	captive
1290	German		3,5	866	16,9	169	12,7	8,9	34,0	41,7	no pyramiding	no deformation	0,18	0,45	0,87	0,98	0,61	boettgeri	2	captive
1291	German		3,0	1028	16,1	161	12,6	9,5	34,5	43,2	pyramiding	no deformation	0,25	0,53	1,02	1,03	0,69	boettgeri	3	captive
1292	German		3,5	1127	17,3	17	13,7	9,3	37,0	44,5	no pyramiding	no deformation	0,22	0,51	0,98	12,75	0,68	boettgeri	3	captive
1293	German		3,0	1395	17,7	177	14,1	10,1	39,8	47,8	no pyramiding	no deformation	0,25	0,55	1,06	1,03	0,73	boettgeri	3	captive
1294	German		2,5	1593	19,3	193	14,9	9,8	41,0	50,7	no pyramiding	no deformation	0,22	0,57	1,08	1,02	0,77	boettgeri	3	captive
1295	German		3,0	439	13,0	130	9,7	6,4	26,2	31,5	no pyramiding	no deformation	0,20	0,54	1,04	0,99	0,53	boettgeri	0	captive
1296	German		2,0	1304	18,3	18	13,5	9,0	36,4	47,3	no pyramiding	no deformation	0,21	0,59	1,12	10,12	0,76	boettgeri	2	captive
1297	German		3,5	314	12,7	127	11,2	7,4	32,0	36,5	no pyramiding	no deformation	0,15	0,30	0,57	0,94	0,27	boettgeri	3	captive
1298	German		3,0	921	15,1	151	11,0	6,1	33,9	40,0	no pyramiding	no deformation	0,27	0,91	1,74	1,04	0,68	boettgeri		captive
1299	German		3,0	2008	25,0	250	16,5	9,5	43,5	57,5	no pyramiding	no deformation	0,13	0,51	0,98	0,96	0,80	boettgeri	4	captive
1300	German		3,0	458	13,1	131	10,5	6,6	27,6	36,5	no pyramiding	no deformation	0,20	0,50	0,96	0,99	0,45	boettgeri	2	captive
1301	German		3,5	1418	21,0	210	13,7	8,1	38,0	45,3	pyramiding	deformation	0,15	0,61	1,16	0,97	0,82	boettgeri	3	captive
1302	German		3,0	1277	22,0	220	12,0	8,3	36,0	51,0	pyramiding	deformation	0,12	0,58	1,11	0,94	0,70	boettgeri	1	captive
1303	German		3,0	1733	26,0	260	14,5	14,0	39,5	60,0	no pyramiding	no deformation	0,10	0,33	0,63	0,93	0,73	boettgeri	5	captive
1304	German		3,0	457	13,4	134	10,0	5,0	28,0	33,0	no pyramiding	no deformation	0,19	0,68	1,30	0,98	0,49	boettgeri	0	captive
1305	German		3,0	205	10,0	100	8,0	5,0	22,7	27,0	no pyramiding	no deformation	0,21	0,51	0,98	0,98	0,33	boettgeri		captive
1306	German		3,0	341	11,5	115	9,1	7,3	26,7	31,5	pyramiding	deformation	0,22	0,45	0,85	1,00	0,41	boettgeri	0	captive
1307	German		3,5	699	14,9	149	11,9	8,1	33,8	41,0	no pyramiding	no deformation	0,21	0,49	0,93	1,00	0,50	boettgeri	1	captive
1308	German		3,0	1417	20,0	200	15,2	10,4	39,4	47,2	no pyramiding	no deformation	0,18	0,45	0,86	0,99	0,76	boettgeri	5	captive
