# Supplementary material for: Body condition scoring facilitates healthcare monitoring in Hermann’s Tortoises (Testudo hermanni ssp.)
Source: PLoS One. 2024 Apr 18;19(4):e0301892. doi: 10.1371/journal.pone.0301892 (PMC11025769; doi:10.1371/journal.pone.0301892)
Supplement: S2 File — (PDF) [file pone.0301892.s002.pdf]

Medizinische Kleintierklinik • Veterinärstr. 13 • 80539 München

Ethik-Kommission der veterinärmedizinischen Fakultät der LMU München

Petra Kölle

Priv.-Doz., Dr. med. vet., Dr. med. vet. habil.
Fachtierärztin für Fische
Fachtierärztin für Reptilien
Teilgebietsbezeichnung Zierfische
Zusatzbezeichung Ernährungsberatung (Kleintiere)

Telefon +49 (0)89-2180-2650
p.koelle@medizinische-kleintierklinik.de

München, 09.07.2020

Antrag auf Zustimmung der Ethikkommission
für die Durchführung der Dissertation mit dem vorläufigen Titel:

„Body Condition Score und Wachstumskurven bei Landschildkröten anhand der Spezies Griechische Landschildkröte *(Testudo hermanni boettgeri),* Maurische Landschildkröte (*Testudo graeca*) und Russische Landschildkröte (*Testudo horsfieldii*)"

Antragssteller: Julia Frankenberger und PD Dr. Petra Kölle
Anfangsdatum: 01.08.2020
Enddatum: 31.12.2024

Einführung

Zur Beurteilung der Gesundheit eines Tieres ist die Erfassung des Ernährungszustandes (BCS = Body Condition Score) ein unverzichtbarer Bestandteil der allgemeinen Untersuchung auch nach Good Veterinary Practice. Schildkröten stellen hier mit ihrem Panzer und der Verteilung des Fettgewebes eine besondere Herausforderung dar. Die bisherige Erfassung der Körperkondition basiert überwiegend auf einer Korrelation von Körpergewicht zu Panzerlänge (Jackson 1980, Jacobson 1993, Hailey 2000, Willemsen 2002).
In bestehenden Studien gab es jedoch regionale Unterschiede in Bezug auf die Körpergröße und nach Jacobson (1993) gibt es mehrere Faktoren, die die Aussagekraft der Korrelation von Körpergewicht zur Panzerlänge einschränken. Die weitere Fragestellung bezieht sich somit darauf, ob die bisherige Messmethodik zur Erhebung der Körperkondition, die Korrelation von Panzerlänge zu Gewicht, mit weiteren Messpunkten, wie der Panzerhöhe, - breite und des Body Condition Scores für die Praxis und weitere Studien sinnvoll ergänzt werden können. Mithilfe der Panzermessungen können zusätzlich zum Wachstum auch die Körperproportionen der Tiere erfasst werden. Interessant ist dies insbesondere im Hinblick auf die unterschiedlichen Panzerformen der Spezies untereinander, sowie der geschlechtsspezifischen Unterschiede besonders bei *T.horsfieldii* (Bonnet 2001).

Da die Wachstumsrate bei Reptilien abhängig von Fütterung und Umgebungstemperaturen ist und somit in Menschenhand vom Halter direkt beeinflusst wird, ist eine genaue Kenntnis des Wachstumsverlaufes von Schildkröten jedoch von entscheidender Bedeutung für die artgerechte Haltung. Bei übermäßiger Fütterung und dadurch bedingtem zu schnellem Wachstum können schwerwiegende gesundheitliche Probleme wie Adipositas, Leberverfettung, präovulatorische Legenot und irreversible Deformierungen des Panzers die Folge sein.

Bezüglich des Wachstums stellt sich die Frage, inwieweit die Daten wildlebender Tiere (Bonnet 2001, Hailey 2000, Willemsen 2002, Lapid 2005, Zivkov 2007, Ritz 2012) tatsächlich auf Tiere übertragen werden können, die in Menschenobhut aufgezogen wurden. So zeigte sich mehrfach ein saisonaler Unterschied im „Peak", also der höchsten Körperkondition, sowie in der Wachstumsgeschwindigkeit der Tiere.

Medizinische Kleintierklinik
Zentrum für klinische Tiermedizin
Ludwig-Maximilians-Universität München
Vorstand: Prof. Dr. Katrin Hartmann
USt-IdNr. DE 811 205 325

Öffentliche Verkehrsmittel:
U-Bahn Linie 3 und 6
Haltestelle „Universität"
Telefon: +49 (0)89-2180-2650
Telefax: +49 (0)89-2180-6240

Verwaltung: Königinstr. 16
Klinik: Veterinärstr. 13
80539 München
info@medizinische-kleintierklinik.de
www.medizinische-kleintierklinik.de

Mehr Daten zu Schildkröten als Heimtiere werden benötigt, um einen Bereich definieren zu können, in dem die Körperkondition bzw. BCS und den Wachstumsverlauf als „normal" einstufen zu können. Ein besonderer Fokus liegt hierbei auf der Korrelation des Wachstums mit dem bekannten Alter der Tiere in menschlicher Obhut, da aufgrund Artenschutzrichtlinien jedes Tier entsprechende Papiere besitzen muss, in denen auch das Schlupfdatum angegeben ist. Dies ist ein Faktor, der bei wildlebenden Kolonien oft nur anhand von Wachstumsringen geschätzt werden kann, eine Methodik, die jedoch nicht immer verlässlich funktioniert (Bertolero 2005). Das erste Ziel der Studie besteht darin, Daten von Tieren in menschlicher Obhut zu erhalten und diese mit bisherigen Messungen bezüglich des BCS bei Schildkröten anderer Spezies (Lamberski 2013, Rawski 2014) sowie von Gewicht und Panzerlänge wildlebender Tiere (Bonnet 2001, Hailey 2000, Willemsen 2002, Lapid 2005, Zivkov 2007, Ritz 2012) der jeweiligen Spezies zu vergleichen, sowie festzustellen, ob Speziesunterschiede vorhanden sind.

Studiendesign

Für die Studie werden gemäß der t-test poweranalyse von Dr. Yury Zablotski pro Spezies mindestens 85 Tiere pro Altersgruppe (juvenil, subadult, adult) sowie bei adulten Tieren pro Geschlecht (männlich, weiblich) benötigt, um eine 90% Power bei einer mittleren Effektstärke von 0,5 zu erreichen. Grundsätzlich ist die genaue Anzahl im Vorraus schwierig festzulegen, da jeder Händler eine unterschiedliche Anzahl an Tieren vor Ort hat, die in diesen Zeitraum vermessen werden können. Darum sollte von mindestens 100 Tieren pro Altersgruppe und Geschlecht, d.h. insgesamt von 400 Tieren pro Spezies ausgegangen werden.

Da für die Subspezies von Testudo hermanni (T. h. hermanni und Testudo h. boettgeri) in vorangegangenen Studien Unterschiede in der Körperkondition festgestellt wurden, werden nur Tiere der Spezies Testudo *hermanni boettgeri* berücksichtigt (Willemsen 2002). Ein Body Condition Score wird im Vorfeld in einer 5-Punkte Skala (1 = kachektisch, 3 = idealer Ernährungszustand, 5 = adipös) anhand der Ausprägung von Nacken-, Bein- und Schwanzmuskulatur erstellt.

Für die Studientiere wird zunächst bei Reptiliengroßhändlern und Züchtern angefragt, mit dem Vorteil, dass größere Tiergruppen einheitlich gehalten und gefüttert werden, sowie Herkunft und Alter der Tiere bekannt ist. Sollten sich auf

diese Weise nicht genügend Probanden generieren lassen, wird auf Tiere in Auffangstationen zurückgegriffen.

Nach Erhebung der Anamnese bezüglich Alter, Herkunft, Fütterung und Geschlecht werden die einzelnen Tiere mindestens 12h nach der letzten Fütterung gewogen, ihre Panzerlänge, -breite und -höhe mithilfe eines Maßbandes und eines Messschiebers gemessen sowie ihr Body Condition Score erhoben. Das Handling der Tiere erfolgt dabei möglichst schonend, sodass eine Verfälschung des Ergebnisses durch spontanen Urin- oder Kotabsatz und Stress für die Tiere so gering wie möglich gehalten wird.

Die Statistik wird mit Hilfe des Statistikers Dr. Yury Zablotski durchgeführt.

Da das reine Handling, Vermessen und Wiegen in der Regel bei den Verkaufs- und als Heimtieren gehaltenen Tieren, sowie bei den Tieren in den Auffangstationen regelmäßig durchgeführt wird (dies ist inklusive Fotos auch für die regelmäßige Meldung bei den Artenschutzbehörden erforderlich), stellt die Durchführung dieser Tätigkeiten zur Datenerhebung keine übermäßige Beanspruchung der Tiere im Sinne eines anzeigepflichtigen oder meldepflichtigen Tierversuches dar.

Mit freundlichen Grüßen,

Dr. med.vet. Petra Kölle
Priv.-Doz., Dr. med. vet., Dr. med. vet. habil.
Fachtierärztin für Fische
Fachtierärztin für Reptilien
Teilgebietsbezeichnung Zierfische
Zusatzbezeichnung Ernährungsberatung (Kleintiere)

Julia Frankenberger
Tierärztin

Literaturverzeichnis

BERTOLERO, A., CARRETERO, MIGUEL, LLORENTE, GUSTAVO. (2005). An assessment of the reliability of growth rings counts for age determination in the Hermann's Tortoise Testudo hermanni. Amphibia-Reptilia. 26. 17-23. 10.1163/1568538053693198

BONNET, X., LAGARDE, F., HENEN, B. T., CORBIN, J., NAGY, K. A., NAULLEAU, G., BALHOUL, K., CHASTEL, O., LEGRAND, A. & CAMBAG, R. (2001). Sexual dimorphism in steppe tortoises (*Testudo horsfieldii*): influence of the environment and sexual selection on body shape and mobility. *Biological Journal of the Linnean Society* 72, 357-372

CARROLL C.L., HUNTINGTON P.J. (1988). Body condition scoring and weight estimation of horses. Equine Vet. J., 20: 41–45

EDMONSON A.J., LEAN I.J., WEAVER L.D., FARVER T., WEBSTER G. (1989). A body condition scoring chart for Holstein dairy cows. J. Dairy Sci., 72: 68–78

FRANZ, R., HUMMEL, J., MÜLLER, D. W.H., BAUERT, M., HATT, J. M., CLAUSS, M.: Herbivorous reptiles and body mass: Effects on food intake, digesta retention, digestibility and gut capacity, and a comparison with mammals, Comparative Biochemistry and Physiology Part A: Molecular & Integrative Physiology, Volume 158, Issue 1, 2011, Pages 94-101, ISSN 1095-6433, https://doi.org/10.1016/j.cbpa.2010.09.007.

FURRER, S.C., HATT, J.M., SNELL, H., MARQUEZ, C., HONEGGER, R.E., RÜBEL, A. (2004). Comparative study on the growth of juvenile galapagos giant tortoises (*Geochelone nigra*) at the Charles Darwin Research Station (Galapagos Islands, Ecuador) and Zoo Zurich (Zurich, Switzerland). *Zoo Biology* 23, 177-183

GERMAN A.J. (2006). The growing problem of obesity in dogs and cats. J. Nutr., 136: 1940–1946.

GERLACH, JUSTIN (2010) Effects of diet on the systematic utility of the tortoise carapace, African Journal of Herpetology, 53:1, 77-85, DOI: 10.1080/21564574.2004.9635499

GIMMEL A, ÖFNER S, LIESEGANG A. (2020) Body condition scoring (BCS) in corn snakes (Pantherophis guttatus) and comparison to pre-existing body condition index (BCI) for snakes. J Anim Physiol Anim Nutr. 2020;00:1–5. https://doi.org/10.1111/jpn.13291

HAILEY A. (2000). Assessing body mass condition in the tortoise *Testudo hermanni*. Herpetol. J., 10: 57–6

HAILEY, A., COULSON, I.M. (1999). The growth pattern of the African tortoise *Geochelone pardalis* and other chelonians. *Canadian Journal of Zoology* 77, 181-193

HAILEY, A., LAMBERT, M.R.K. (2002). Comparative growth patterns in Afrotropical giant tortoises (Reptilia Testudinae). *Tropical Zoology* 15, 121-13

HENNEKE, D. R., POTTER, G. D., KREIDER, J. L., & YEATES, B. F. (1983). Relationship between condition score, physical measurements and body fat percentage in mares. Equine Veterinary Journal, 15(4), 371–372. https://doi.org/10.1111/j.2042-3306.1983.tb018 26.x

JACKSON, C., THOMAS H. TROTTER, TROTTER, J., & MARY W. TROTTER. (1978). Further Observations of Growth and Sexual Maturity in Captive Desert Tortoises (Reptilia: Testudines). *Herpetologica, 34*(2), 225-227

JACKSON O.F. (1980). Weight and measurement data on tortoises (*Testudo graeca* and *Testudo hermanni*) and their relationship to health. J. Small Anim. Prac., 2: 409–416

JACOBSON E., WEINSTEIN M., BERRY K., HARDENBROOK B., TOMLINSON C., FREITAS D. (1993). Problems with using weight versus carapace length relationships to assess tortoise health. Vet. Rec., 132: 222–223

JONES T.T., HASTINGS M.D., BOSTROM B.L., PAUL Y D., JONES D.R. (2011). Growth of captive leatherback turtles, *Dermochelys coriacea*, with inferences on growth in the wild: Implications for population decline and recovery. J. Exp. Mar. Biol. Ecol., 399: 84–92.

KÖLLE, P. (Hrsg.) (2009): Die Schildkröte. Heimtier und Patient. 1.Aufl., Enke, Stuttgart.

LAFLAMME, D. R. P. C. (1997). Development and validation of a body condition score system for dogs.: A clinical tool. Canine Practitioner, 22, 10 –1 5.

LAMBERSKI, N. (2013). Body condition scores for desert tortoises. [PDF file]. Retrieved from https ://www.fws.gov/nevad a/desert_torto ise/documents/reports/2013/asses s/Desert-Torto ise-BCS-2013-lambe rski-po.pdf

LAPID, R.H., NIR, I., ROBINZON, B. (2005). Growth and body composition in captive *Testudo graeca terrestris* fed with a high-energy diet. *Applied Herpetology* 2, 201-20

MADER R. (2006). Reptile Medicine and Surgery. St. Louis, USA, Elsevier Saunders, 2nd ed., pp. 251–294.

RAWSKI, M., & JÓZEFIAK, D. (2014). Body condition scoring and obesity in captive African side-neck turtles (Pelomedusidae). Annals of Animal Science, 14(3), 573–584. https://doi.org/10.2478/aoas-2014-0037

RITZ, J., CLAUSS, M., STREICH, W. J., HATT, J .M. (2012): Variation in Growth and Potentially Associated Health Status in Hermann's and Spur-Thighed Tortoise (*Testudo hermanni* and *Testudo graeca*), https://doi.org/10.1002/zoo.21002

RITZ J., HAMMER C., CLAUSS M. (2010). Body size development of captive and free-ranging leopard tortoises (*Geochelone pardalis*). Zoo Biol., 29: 517–525

THOMSON, J. A., BURKHOLDER, D., HEITHAUS, M. R., & DILL, L. M. (2009). Validation of a rapid visual-assessment technique for categorizing the body condition of green turtles (Chelonia mydas) in the field. Copeia, 2009(2), 251–255. https://doi.org/10.1643/CE-07-227

WILLEMSEN R.E., HAILEY A. (2002). Body mass condition in Greek tortoises: Regional and interspecific variation. Herpetol. J., 12: 105–114

WILLEMSEN R.E., HAILEY A., LONGEPIERRE S., GRENOT C. (2002). Body mass condition and management of captive European tortoises. Herpetol. J., 12: 115–121

ZWART, P., LAMBRECHTS, L., DE BATIST, P., BIJNENS, B., CLAESSEN, H., MENNES, S., VAN RIEL, C. (1997). Excessive growth of hermann's tortoise (*Testudo hermanni*) and consequences for carapace development – a case report. *Verhandlungsbericht Erkrankungen der Zootiere* 38, 61-64

Ethikkommission der Tierärztlichen Fakultät
Ludwig-Maximilians-Universität München

Prof. Dr. Ralf S. Mueller
Vorsitzender der
Ethikkommission
Tierärztliche Fakultät

der LMU München
Telefon +49 (0)89 2180-2654
Telefax +49 (0)89 2180-6240

r.mueller@medizinische-kleintierklinik.de

An
Julia Frankenberger, PD Dr. Petra Kölle
MKTK des Zentrums für klinische Tiermedizin
LMU München

Studie „Body condition score und Wachstumskurven bei Landschildkröten anhand der Spezies Griechische Landschildkörte (Testudo hermanni boettgeri), Maurische Landschildkröte (Testudo graeca) und Russische Landschildkröte (Testudo horsfieldii)", Aktenzeichen 224-09-07-2020

München, 26.08.2020

Betr.: Antrag an die Ethikkommission zur Überprüfung des Protokolls für die geplante Studie „Body condition score und Wachstumskurven bei Landschildkröten anhand der Spezies Griechische Landschildkörte (Testudo hermanni boettgeri), Maurische Landschildkröte (Testudo graeca) und Russische Landschildkröte (Testudo horsfieldii)"

Sehr geehrte Frau Frankenberger, liebe Julia, liebe Petra,

Ich freue mich, Euch mitteilen zu können, dass seitens der Ethikkommission keine Bedenken gegen die geplante Studie „Body condition score und Wachstumskurven bei Landschildkröten anhand der Spezies Griechische Landschildkörte (Testudo hermanni boettgeri), Maurische Landschildkröte (Testudo graeca) und Russische Landschildkröte (Testudo horsfieldii)" vorliegen und das Protokoll unter der Nummer 224-09-07-2020 genehmigt wurde, wir bräuchten allerdings noch eine Email mit der Angabe, wie lange die Untersuchungen pro Tier dauern.. Diese Genehmigung gilt von 8/2020 bis 8/2024.
Drei der fünf Mitglieder der Kommission haben den Antrag überprüft, ihre speziellen Kommentare sind weiter unten aufgelistet.

Hochachtungsvoll

Ralf Mueller

Prof. Dr. Ralf S. Mueller
Vorsitzender der Ethikkommission
Tierärztliche Fakultät, LMU München

Kommentare:
Etwas unklar bleibt das genaue Studienziel, also ob in dieser Studie Referenzwerte, also Normalwerte erfasst werden sollen. In diesem Fall wäre es eventuell notwendig, „gute" Züchter auszuwählen oder „gute" Händler, die die Schildkröten aus guten Zuchten erwerben. Tiere aus Auffangstationen wären dann eigentlich nicht geeignet.
Es fehlt noch eine Angabe, wie lange die Schildkröten gehandelt werden müssen, bis alle Messparameter erfasst sind.

Kommentar zum Ethikantrag 224-09-07-2020

Von: Julia Frankenberger ([redacted])
An: R.Mueller@medizinische-kleintierklinik.de; p.koelle@medizinische-kleintierklinik.de
Datum: Freitag, 28. August 2020 um 12:33 MESZ

Sehr geehrter Herr Müller, lieber Ralf,

bezüglich der geplanten Studie „Body condition score und Wachstumskurven bei Landschildkröten anhand der Spezies Griechische Landschildkörte (Testudo hermanni boettgeri), Maurische Landschildkröte (Testudo graeca) und Russische Landschildkröte (Testudo horsfieldii)“ und dem Protokoll unter der Nummer 224-09-07-2020 sind hier noch die Anmerkungen:

- Zur Erfassung der Anamnese ist kein Handling der Tiere notwendig. Das Wiegen, Palpieren und Erfassen der Panzermaße beläuft sich auf 5-10 Minuten pro Tier. Diese Angabe beruht auf der Zeit, die für die Messungen an einem entsprechenden Dummy gebraucht wurde.

- Das genaue Ziel der Studie ist die Erfassung von Normwerten bezüglich des Wachstums und der Körperkondition der oben genannten Arten.

- Zur Erfassung von Normalwerten werden Händler und Züchter aufgesucht, die fast nur gesunde Tiere im Bestand haben, um Abweichungen so gering wie möglich zu halten. (Diese können jedoch nicht ausgeschlossen werden)

- Bei Tieren, die aus Auffangstationen vermessen werden, handelt es sich oft um entlaufene Schildkröten aus guter Haltung, wie auch Tiere, die bereits als Jungtiere in die Station kamen, aufgrund des Geschlechts aber nur schwer weitervermittelt werden können. Es ist keine Seltenheit, dass solche Tiere bereits über zehn Jahre in den Stationen (in guter Haltung) gepflegt werden. (Im Gegensatz zu Schlangen, wo die meisten Tiere aus Beschlagnahmungen stammen.)

Mit freundlichen Grüßen,

Julia Frankenberger

Application for approval by the Ethics Committee

for carrying out the dissertation with the provisional title:

"Body condition score and growth curves in tortoises based on the species Hermann's tortoise *(Testudo hermanni boettgeri),* Moorish tortoise *(Testudo graeca*) and Russian tortoise *(Testudo horsfieldii*)"

Applicants: Julia Frankenberger and PD Dr. Petra Kölle
Begin: 01.08.2020
End: 31.12.2024

Introduction

In order to assess the health status of an animal, the Body Condition Score (BCS) is an essential part of the general examination, also according to Good Veterinary Practice.

Tortoises present a particular challenge due to their shell and distribution of adipose tissue. Previous assessments of body condition were based mainly on a correlation between body weight and shell length (Jackson 1980, Jacobson 1993, Hailey 2000, Willemsen 2002).

However, there were regional differences in body size in the existing studies and, according to Jacobson (1993), there are several factors that limit the validity of the correlation between body weight and carapace length. Therefore, the further question is whether the existing method of measuring body condition, the correlation of carapace length with body weight, can be usefully supplemented with other measurements such as carapace height, width and body condition score for practical use and further study. The carapace measurements can be used to determine the body proportions of the animals in addition to their growth. This is particularly interesting in view of the different shell shapes of the species and the sex-specific differences, especially in T. horsfieldii (Bonnet 2001).

Since the growth rate of reptiles is dependent on diet and environmental temperature and is therefore directly influenced by the owner, an accurate knowledge of the growth rate of turtles is essential for proper husbandry. Overfeeding and the resulting rapid growth can lead to serious health problems such as obesity, fatty liver, pre-ovulatory egg loss and irreversible shell deformation.

With regard to growth, it is questionable to what extent data from wild animals (Bonnet 2001, Hailey 2000, Willemsen 2002, Lapid 2005, Zivkov 2007, Ritz 2012) can actually be extrapolated to captive animals. For example, a seasonal difference in the "peak", i.e. the highest body condition, as well as in the growth rate of the animals has been shown several times.

More data on tortoises as pets are needed to define a range in which body condition (BCS) and growth pattern can be considered "normal". A particular focus is on the correlation of growth with the known age of animals in human care, as species protection guidelines require that each animal has appropriate documentation, including date of hatching. This is a factor that can often only be estimated from growth rings in wild colonies, a method that is not always reliable (Bertolero 2005). The first aim of the study is to obtain data from animals in human care and to compare them with previous measurements of BCS in turtles of other species (Lamberski 2013, Rawski 2014) and of weight and carapace length in wild animals (Bonnet 2001, Hailey 2000, Willemsen 2002, Lapid 2005, Zivkov 2007, Ritz 2012) of the respective species, and to determine whether species differences exist.

Study Design

According to Dr. Yury Zablotski's t-test power analysis, the study requires at least 85 animals per species per age group (juvenile, subadult, adult) and for adult animals per sex (male, female) to achieve 90% power with a mean effect size of 0.5. In principle, it is difficult to determine the exact number in advance, as each trader has a different number of animals on site that can be measured during this period. Therefore, a minimum of 100 animals per age group and sex should be assumed, i.e. a total of 400 animals per species.

As previous studies have shown differences in body condition between the subspecies Testudo hermanni (T. h. hermanni and Testudo h. boettgeri), only animals of the species Testudo hermanni boettgeri are considered (Willemsen 2002). A body condition score is determined beforehand on a 5-point scale (1 = cachectic, 3 = ideal nutritional status, 5 = obese) based on the development of neck, leg and tail muscles.

The study animals are first requested from reptile traders and breeders, with the advantage that larger groups of animals are kept and fed uniformly, and the origin and age of the animals are known. If this is not sufficient, animals from rescue centers are used.

After taking a history of age, origin, diet, and sex, individual animals are weighed at least 12 hours after the last feeding, their carapace length, width, and height are measured using a tape measure and calipers, and their body condition score is recorded. The animals are handled as gently as possible to minimize any bias in the results due to spontaneous urination or defecation and stress to the animals.

Statistics are conducted with the assistance of statistician Dr. Yury Zablotski.

Since the pure handling, measuring and weighing of the animals for sale and pets, as well as the animals in the sanctuaries, is usually carried out on a regular basis (this is also necessary for the regular reporting to the species protection authorities, including photos), the performance of these activities to collect data does not constitute excessive stress for the animals in the sense of a notifiable or reportable animal experiment.

Sincerely,

Dr. med.vet. Petra Kölle

Priv.-Doz., Dr. med. vet., Dr. med. vet. habil.

Fachtierärztin für Fische

Fachtierärztin für Reptilien

Teilgebietsbezeichnung Zierfische

Zusatzbezeichnung Ernährungsberatung (Kleintiere)

Julia Frankenberger

Veterinarian

Translation

Decision of the Ethics Committee

Munich, 26 August 2020

Dear Mrs Frankenberger, dear Julia, dear Petra,

I am pleased to inform you that the ethics committee has no objections to the planned study planned study "Body condition score and growth curves in tortoises using the species Greek tortoises (Testudo hermanni boettgeri), Moorish tortoises (Testudo graeca) and Russian tortoise (Testudo horsfieldii)" and the protocol was approved under the number 224-09-07-2020 has been approved, but we would still need an email stating how long the examinations take per animal. This authorisation is valid from 8/2020 to 8/2024.

Three of the five members of the Commission have reviewed the application, their specific comments are listed below.

Sincerely

Prof Dr Ralf S. Mueller
Chairman of the Ethics Committee
Faculty of Veterinary Medicine, LMU Munich

Comments:

The exact aim of the study remains somewhat unclear, i.e. whether reference values, i.e. normal values, are to be recorded in this study. In this case, it might be necessary to select "good" breeders or "good" dealers who purchase the tortoises from good breeders. Animals from rescue centres would then not really be suitable. There is currently a lack of information on how long the tortoises have to be handled until all measurement parameters have been recorded.

Answer:

- There is no need to handle the animals to record the history. Weighing, palpation and recording of carapace measurements takes 5-10 minutes per animal. This estimate is based on the time taken to take measurements on an equivalent dummy.

- The precise aim of the study is to determine the normal values for growth and body condition of the above mentioned species.

- In order to record normal values, dealers and breeders will be visited, who have almost only healthy animals in their possession, in order to keep deviations as small as possible. (However, these cannot be excluded)

- Animals measured from rescue centers are often escaped tortoises from good captivity, as well as animals that came to the center as juveniles but are difficult to rehome due to their sex. It is not uncommon for such animals to have been in captivity for over ten years. (In contrast to snakes, where most animals come from confiscations).
